# Supplementary material for: Chemical modulation of cytosolic BAX homodimer potentiates BAX activation and apoptosis
Source: Nat Commun. 2023 Dec 16;14:8381. doi: 10.1038/s41467-023-44084-3 (PMC10725471; doi:10.1038/s41467-023-44084-3)
Supplement: Supplementary file 1 — Supplementary Information [file 41467_2023_44084_MOESM1_ESM.pdf]

Supplementary Information

Chemical modulation of cytosolic BAX homodimer potentiates BAX activation and apoptosis

Nadege Gitego[1-3], Bogos Agianian[1-3], Oi Wei Mak[1-3], Vasantha Kumar MV[1-3], Emily H. Cheng[4-6], Evripidis Gavathiotis[1-3*]

[1]Department of Biochemistry, Albert Einstein College of Medicine, Bronx NY, USA
[2]Department of Medicine, Albert Einstein College of Medicine, Bronx, NY, USA
[3]Montefiore Einstein Comprehensive Cancer Center, Albert Einstein College of Medicine, Bronx, NY, USA
[4]Human Oncology and Pathogenesis Program, Memorial Sloan Kettering Cancer Center, New York, NY USA
[5]Department of Pathology and Laboratory Medicine, Memorial Sloan Kettering Cancer Center, New York, NY, USA
[6]Weill Cornell Medicine, New York, NY, USA

Contents:

Supplementary Figures 1-16

Supplementary Tables 1

Supplementary Methods

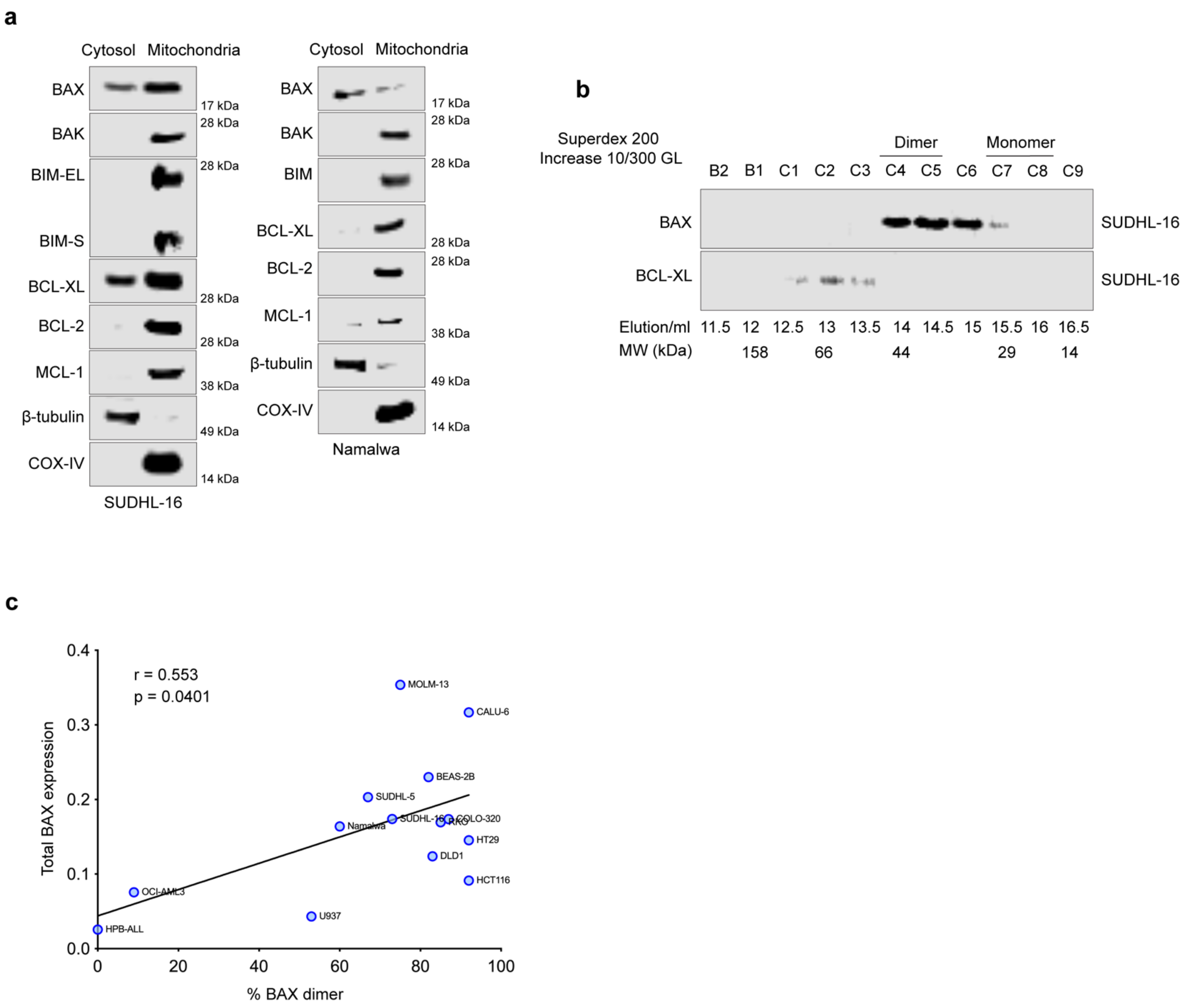

Supplementary Fig. 1. Cellular localization of BCL-2 family proteins and correlation between the cytosolic BAX dimer and total BAX expression.

a Cellular localization of key BCL-2 family members in SUDHL-16 (left) and Namalwa (right). Cytosolic and mitochondrial fractions were separated, and samples analyzed by western blot using indicated antibodies. **b** Size-exclusion chromatography (Superdex 200, HR 10/30 GL) of the cytosolic fraction of SUDHL-16. Elution fractions were analyzed by western blot with BAX and BCL-XL antibodies. The molecular weight as well as the BAX monomer and BAX dimer fractions are indicated. **c** Correlation

between the cytosolic BAX dimer and total BAX expression. Total BAX expression was determined from western blot analysis of whole cell lysates of cell lines (n=14) (Fig. 1a). BAX expression was quantified and normalized against β-actin. The cytosolic BAX dimer and monomer was determined from western blot analysis of elution fractions from the size-exclusion chromatography (Superdex 200, HR 10/30 GL) of cytosolic fractions using a BAX antibody (Fig. 1a). Data in this figure are representative of n=3 independent experiments with similar results. Statistics were obtained using a two-tailed test and Pearson coefficient r: ns, $p \geq 0.05$; *, $p < 0.05$; **, $p < 0.01$; ***, $p < 0.001$; ****, $p < 0.0001$. Source data are provided.

a

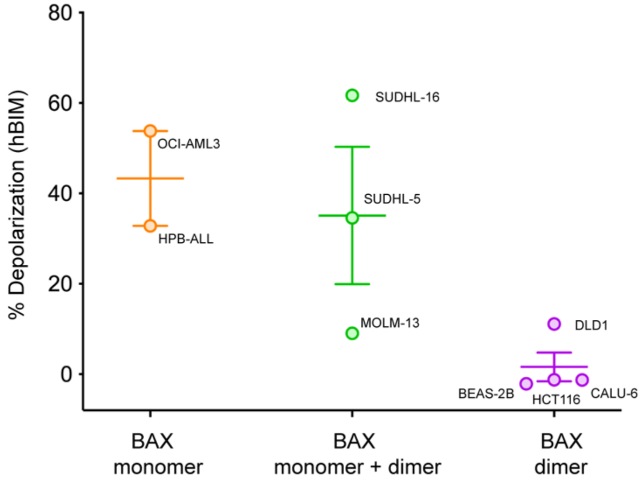

b

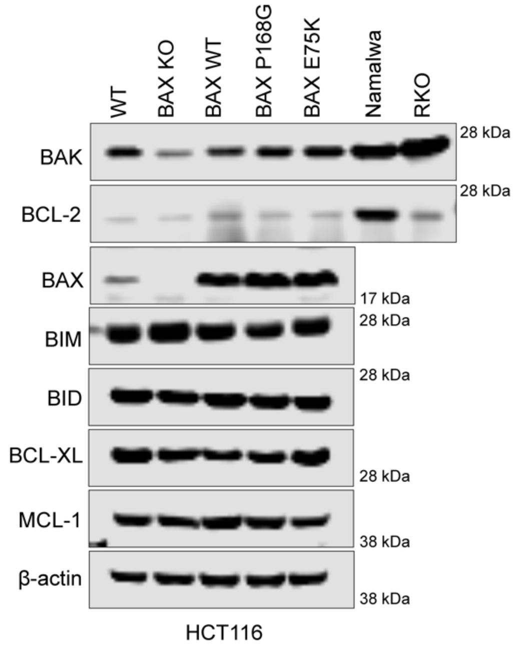

Supplementary Fig. 2. Cells expressing the cytosolic autoinhibited BAX dimer are unprimed to apoptosis and generation of isogenic HCT116 cells expressing cytosolic inactive BAX dimer or BAX monomer.

a Apoptotic priming of a diverse panel of cell lines (n=8) expressing cytosolic BAX dimer or monomer. Mitochondria depolarization was assessed upon 0.1μM BIM BH3 peptide treatment. Cell lines were categorized into cells expressing BAX monomer (monomer > 50%), BAX monomer + dimer (50% > monomer > 25%), and BAX dimer (monomer < 25%). **b** Protein expression of key members of the BCL-2 family protein. Whole lysates of HCT116 WT, HCT116 BAX KO and HCT116 BAX KO reconstituted with BAX WT (cytosolic dimer) or mutants BAX P168G (cytosolic dimer) and BAX E75K (cytosolic monomer), RKO and Namalwa were analyzed with western blot with indicated antibodies. RKO is used as a positive control for BAK and Namalwa for BCL-2. Data in this figure are representative of n=3 independent experiments with similar results. Source data are provided.

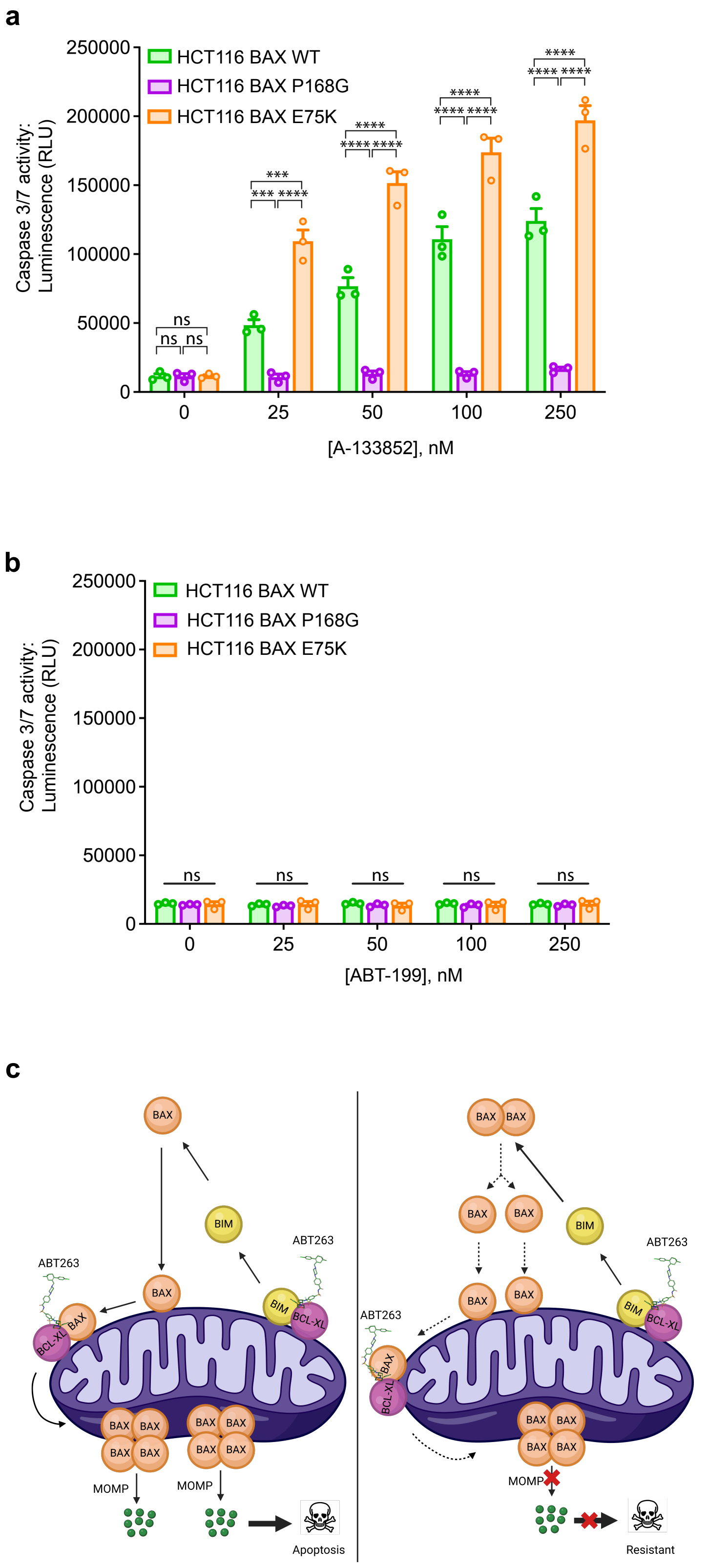
a
Caspase 3/7 activity:
Luminescence (RLU)
250000
200000
150000
100000
50000
0
HCT116 BAX WT
HCT116 BAX P168G
HCT116 BAX E75K
ns
ns ns
***
*** ****
****
**** ****
****
**** ****
****
**** ****
0
25
50
100
250
[A-133852], nM
b
Caspase 3/7 activity:
Luminescence (RLU)
250000
200000
150000
100000
50000
0
HCT116 BAX WT
HCT116 BAX P168G
HCT116 BAX E75K
ns
ns
ns
ns
ns
0
25
50
100
250
[ABT-199], nM
c
BAX
BIM
ABT263
BAX
BCL-XL
BAX
BIM
BCL-XL
ABT263
BAX BAX
BAX BAX
BAX BAX
BAX BAX
MOMP
MOMP
Apoptosis
BAX BAX
BAX
BAX
BIM
ABT263
BAX
BAX
ABT263
BIM
BCL-XL
BAX
BCL-XL
BAX BAX
BAX BAX
MOMP
Resistant

Supplementary Fig. 3. The cytosolic autoinhibited BAX dimer promotes resistance to apoptosis.

a Caspase-3/7 activity of HCT116 BAX KO reconstituted with BAX WT (cytosolic dimer) or mutants, BAX P168G (cytosolic dimer) and BAX E75K (cytosolic monomer) treated with a titration of a BCL-XL specific inhibitor, A-133852 for 2 hrs. **b** Caspase-3/7 activity of HCT116 BAX KO reconstituted with BAX WT (cytosolic dimer) or mutants, BAX P168G (cytosolic dimer) and BAX E75K (cytosolic monomer) treated with a titration of a BCL-2 specific inhibitor, venetoclax (ABT199) for 6 hrs. **c** Schematic showing resistance to apoptosis induction by ABT-263 in cells with cytosolic BAX dimer due to reduced BAX activation by released BIM (right). In contrast, cells with cytosolic BAX monomer can undergo apoptosis due to increased activation of the BAX monomer by BIM (left). This figure was created with BioRender.com. (**a,b**) are mean ± SEM of n=3 independent experiments. Statistics were obtained using two-way ANOVA: ns, $p \geq 0.05$; *, $p<0.05$; **, $p<0.01$; ***, $p<0.001$; ****, $p<0.0001$. Source data are provided.

a

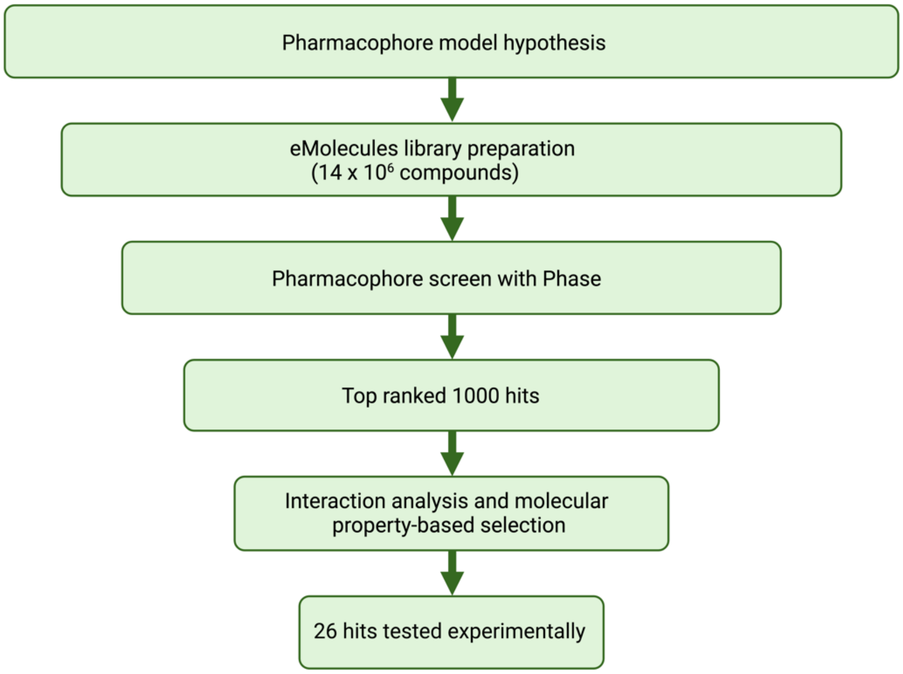

b

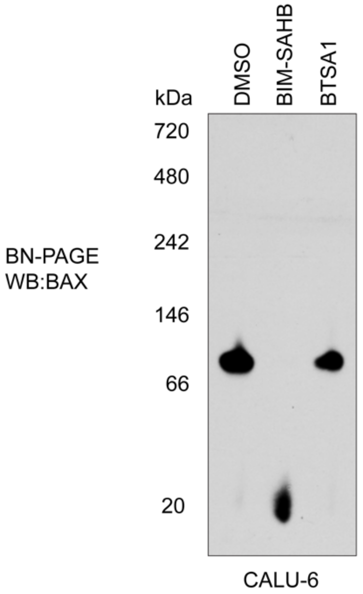

Supplementary Fig. 4. Pharmacophore-based high-throughput *in-silico* screen and biochemical assay

a A library of ~14 million compounds was screened against our pharmacophore model hypothesis using Phase (Schrödinger, LLC). The top 1000 hits were clustered for chemical similarity based on the

Tanimoto fingerprinting method. The top 300 molecules from the similarity clustering were visually inspected for interactions with BAX and analysis of their molecular properties, resulting in selection of top 26 hits for experimental testing. **b** BN-PAGE of CALU-6 cytosolic fractions. The cytosolic fractions were treated with DMSO or BTSA1 (50 μM) for 1 hr at 30°C or BIM-SAHB (50 uM) for 1hr on ice. The samples were analyzed by BN-PAGE and immunoblotted for BAX. (**b**) is a representative of at least n=2 independent experiments with similar results. Source data are provided.

a

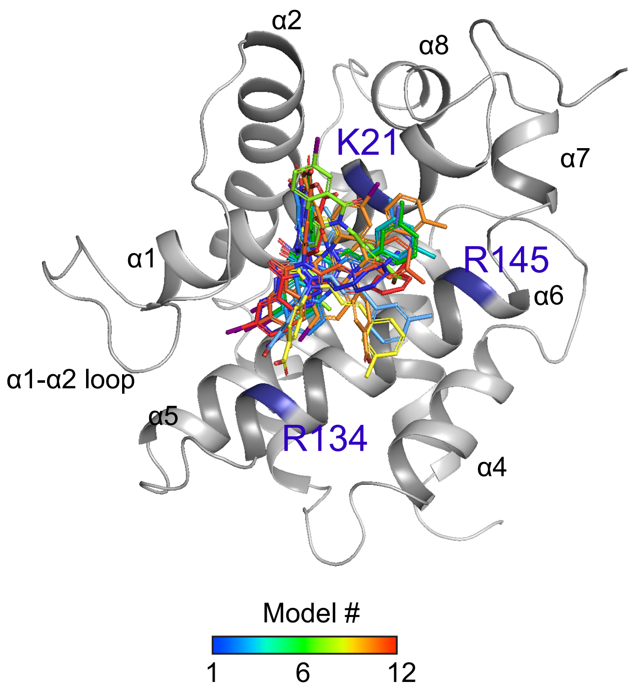

b

Model #	Gscore/ kcal.mol^{-1}	IFDScore/ kcal.mol^{-1}
1	-3.758	-372.97
2	-3.211	-372.53
3	-3.534	-372.27
4	-3.200	-371.57
5	-1.945	-371.27
6	-2.085	-370.99
7	-2.542	-370.72
8	-2.699	-370.58
9	-1.652	-370.26
10	-1.525	-370.21
11	-0.931	-369.74
12	0.096	-367.79

c

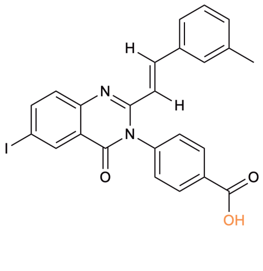

BDM19

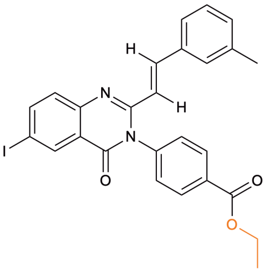

BDM19.1

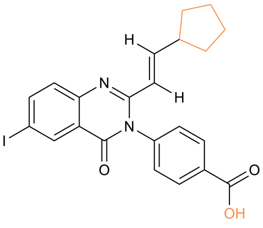

BDM19.2

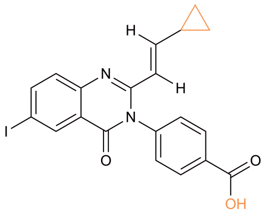

BDM19.3

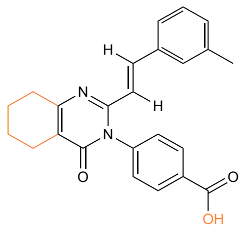

BDM19.4

d

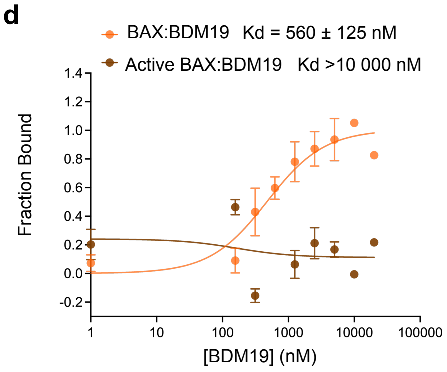

Supplementary Fig. 5. BDM19 binds at the N-terminal trigger site and interacts with K21, Q28, R134 and R145.

a Induced-fit docking of BDM19 into the inactive BAX solution structure (PDB: 1F16) resulting in 12 poses of BMD19 at a site centered around K21, Q28, R134 and R145 residues. **b** The Glide docking and Induced Fit Docking scores for the 12 poses shown in (**a**). **c** Chemical structure of BDM19 and its analogs BDM19.1, BDM19.2, BDM19.3 and BDM19.4. **d** Microscale thermophoresis direct binding of BDM19 to BAX 4C (orange) in inactive conformation and active BAX conformation (brown). Data are mean ± SEM from n=3 independent experiments. Source data are provided.

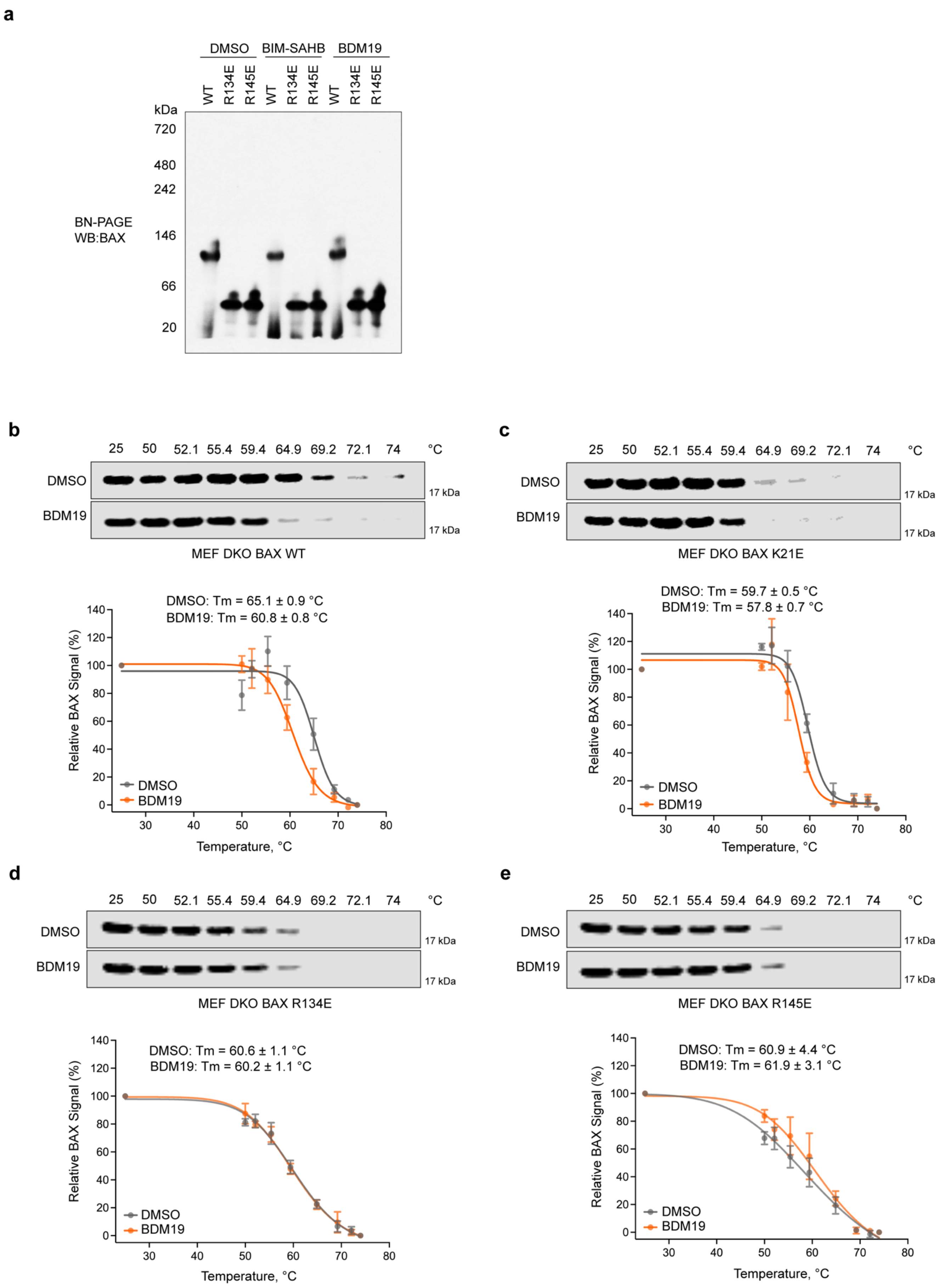

a
DMSO
BIM-SAHB
BDM19
WT
R134E
R145E
kDa
720
480
242
146
66
20
BN-PAGE
WB:BAX
b
25 50 52.1 55.4 59.4 64.9 69.2 72.1 74 °C
DMSO
BDM19
17 kDa
MEF DKO BAX WT
DMSO: Tm = 65.1 ± 0.9 °C
BDM19: Tm = 60.8 ± 0.8 °C
Relative BAX Signal (%)
Temperature, °C
c
MEF DKO BAX K21E
DMSO: Tm = 59.7 ± 0.5 °C
BDM19: Tm = 57.8 ± 0.7 °C
d
MEF DKO BAX R134E
DMSO: Tm = 60.6 ± 1.1 °C
BDM19: Tm = 60.2 ± 1.1 °C
e
MEF DKO BAX R145E
DMSO: Tm = 60.9 ± 4.4 °C
BDM19: Tm = 61.9 ± 3.1 °C

Supplementary Fig. 6. BDM19 cellular activity is dependent on K21, R134 and R145 engagement. **a** BN-Page of MEF BAX BAK DKO reconstituted with BAX WT or BAX mutants R134E and R145E. Cytosolic fractions were treated with DMSO, 50 μM BIM-SAHB or 50 μM BDM19 for 45min at 37°C or on ice for BIM-SAHB. The samples were analyzed by BN-PAGE and immunoblotted for BAX. (**a**) is a representative of at least n=2 independent experiments with similar results. Source data is provided. **b-e** Cellular BAX engagement assay in MEF BAX BAK DKO reconstituted with (**b**) BAX WT or BAX mutants (**c**) K21E, (**d**) R134E and (**e**) R145E. Cells were treated with vehicle (DMSO) or 60 μM of BDM19 for 1 hr at room temperature (RT). Blots (top) are representative of n=3 independent experiments. Melting curves for BAX (bottom) were generated by densitometric analysis. (**b-e**) Data are mean ± SEM from n=3 independent experiments. Source data are provided.

a

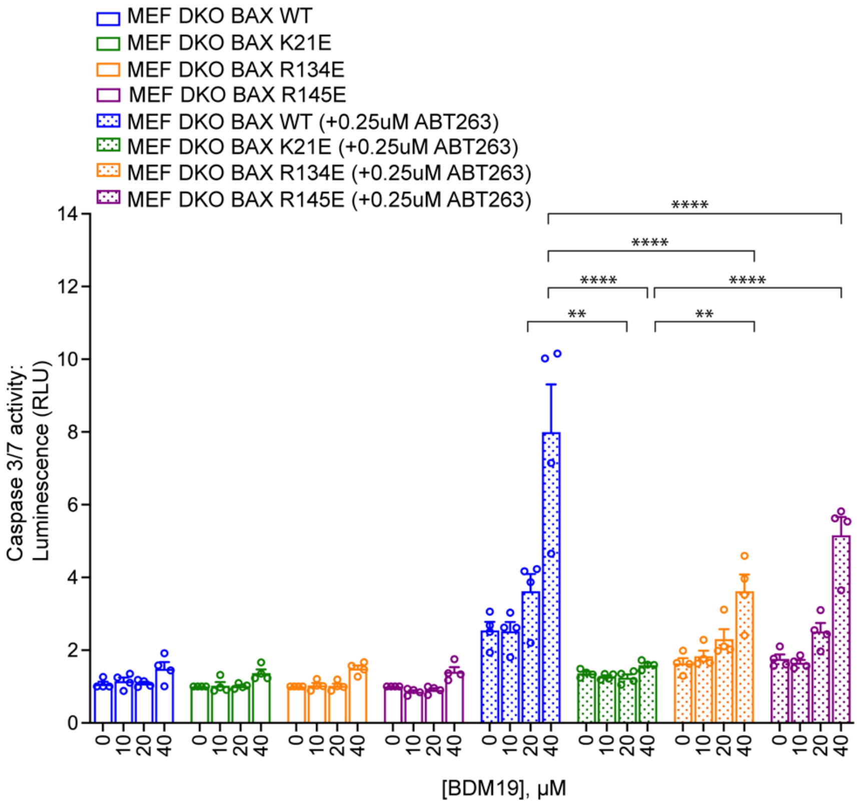

b

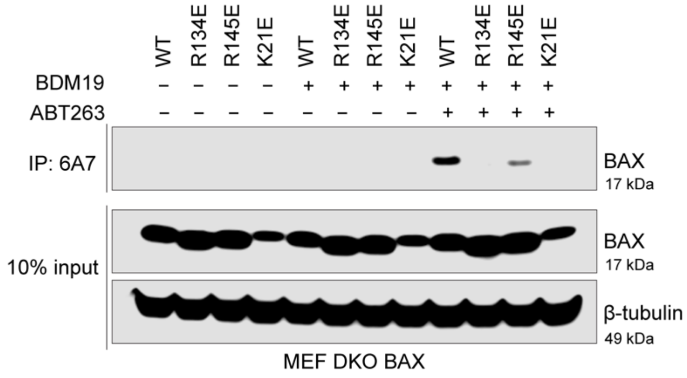

Supplementary Fig. 7. BDM19 apoptotic activity is dependent on K21, R134 and R145 engagement.

a Caspase 3/7 activity of MEF BAX BAK DKO reconstituted with BAX WT or mutants K21E, R134E and R145E treated with a titration of BDM19 and 0.25 μM ABT263 at 6 hrs. **b** 6A7 co-immunoprecipitation of active BAX in MEF BAX BAK DKO reconstituted with BAX WT or mutants

R134E, R145E and K21E. Cells were treated with DMSO, 40 μM BDM19 or a combination of 40 μM BDM19 and 100 nM ABT263 for 2hrs. (**b**) Data are mean ± SEM of n=3 independent experiments. Source data are provided.

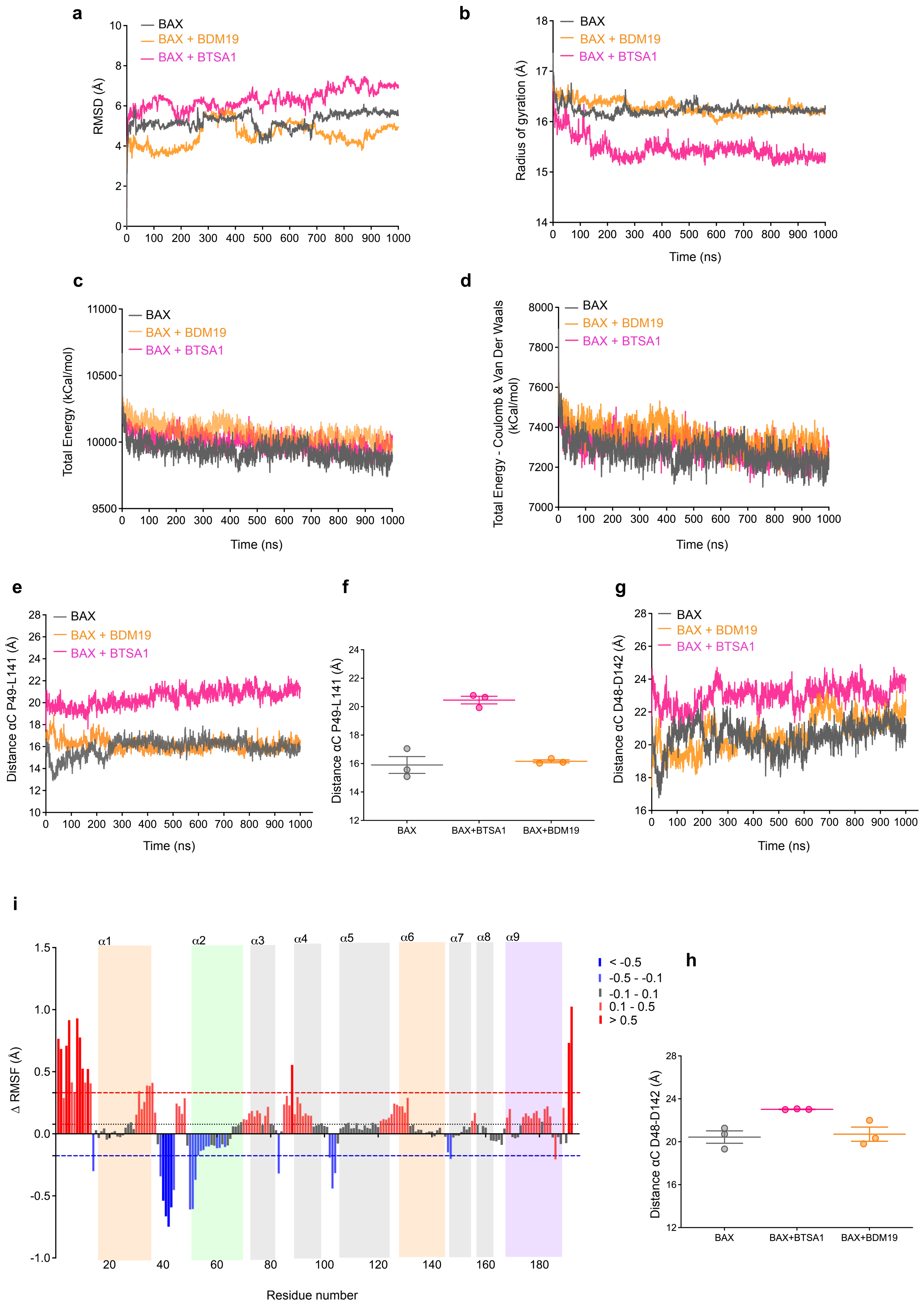
a
BAX
BAX + BDM19
BAX + BTSA1
RMSD (Å)
b
Radius of gyration (Å)
Time (ns)
c
Total Energy (kCal/mol)
d
Total Energy - Coulomb & Van Der Waals
(kCal/mol)
e
Distance αC P49-L141 (Å)
f
BAX
BAX+BTSA1
BAX+BDM19
g
Distance αC D48-D142 (Å)
i
Δ RMSF (Å)
Residue number
α1
α2
α3
α4
α5
α6
α7
α8
α9
< -0.5
-0.5 - -0.1
-0.1 - 0.1
0.1 - 0.5
> 0.5
h

Supplementary Fig. 8. Molecular dynamics (MD) simulations of unbound BAX, the BAX-BDM19 and BAX-BTSA1 complex with distance analysis of unbound BAX, the BAX-BDM19 and the BAX-BTSA1 changes in α1-α2 loop.

a Root-mean square deviation (RMSD) of atoms in the α-carbon of BAX for unbound BAX, BAX-BDM19, BAX-BTSA1 MD simulations is plotted with respect to time. Mean RMSD from n=3 BAX, BAX-BDM19 and BAX-BTSA1 MD simulations. **b** Radius of gyration of BAX, BAX-BDM19 and BAX-BTSA1 is plotted with respect to time. Mean radius of gyration from n=3 BAX, BAX-BDM19 and BAX-BTSA1 MD simulations. **c-d** Energy of simulations of BAX, BAX-BDM19 and BAX-BTSA1. (**c**) represents total energy and (**d**) total energy from Coulomb and Van Der Waals plotted against time. Mean total energy from n=3 BAX, BAX-BDM19 and BAX-BTSA1 simulations. **e-h** Distances in MD simulations between (**e**, **f**) P49 and L141, and (**g**, **h**) D48 and D142. Distances are calculated from α-carbons and are represented as distance with respect to time (**e**, **g**) or as the average of all distances over 1000 ns (**f**, **h**). (**e**-**h**) Data are mean ± SEM from n=3 independent MD simulations. **i** Change in root-mean square fluctuation (RMSF) of BAX-BDM19 and unbound BAX is plotted with respect to BAX residue number. The color gradient is representative of change in RMSF, with blue and red corresponding to decrease and increase in RMSF, respectively. Difference of mean RMSF of unbound BAX from BAX-BDM19 complex. Mean RMSF from n=3 simulations. The average change in RMSF is represented by a black dotted line and average ± SD with red and blue dashed lines respectively. The N-terminal trigger site, BH3 domain, canonical site and the C-terminal transmembrane domain are highlighted in orange, green, gray, and violet, respectively. Data represent mean n=3 simulations for both unbound BAX and BAX-BDM19 complex MD simulations. Source data are provided.

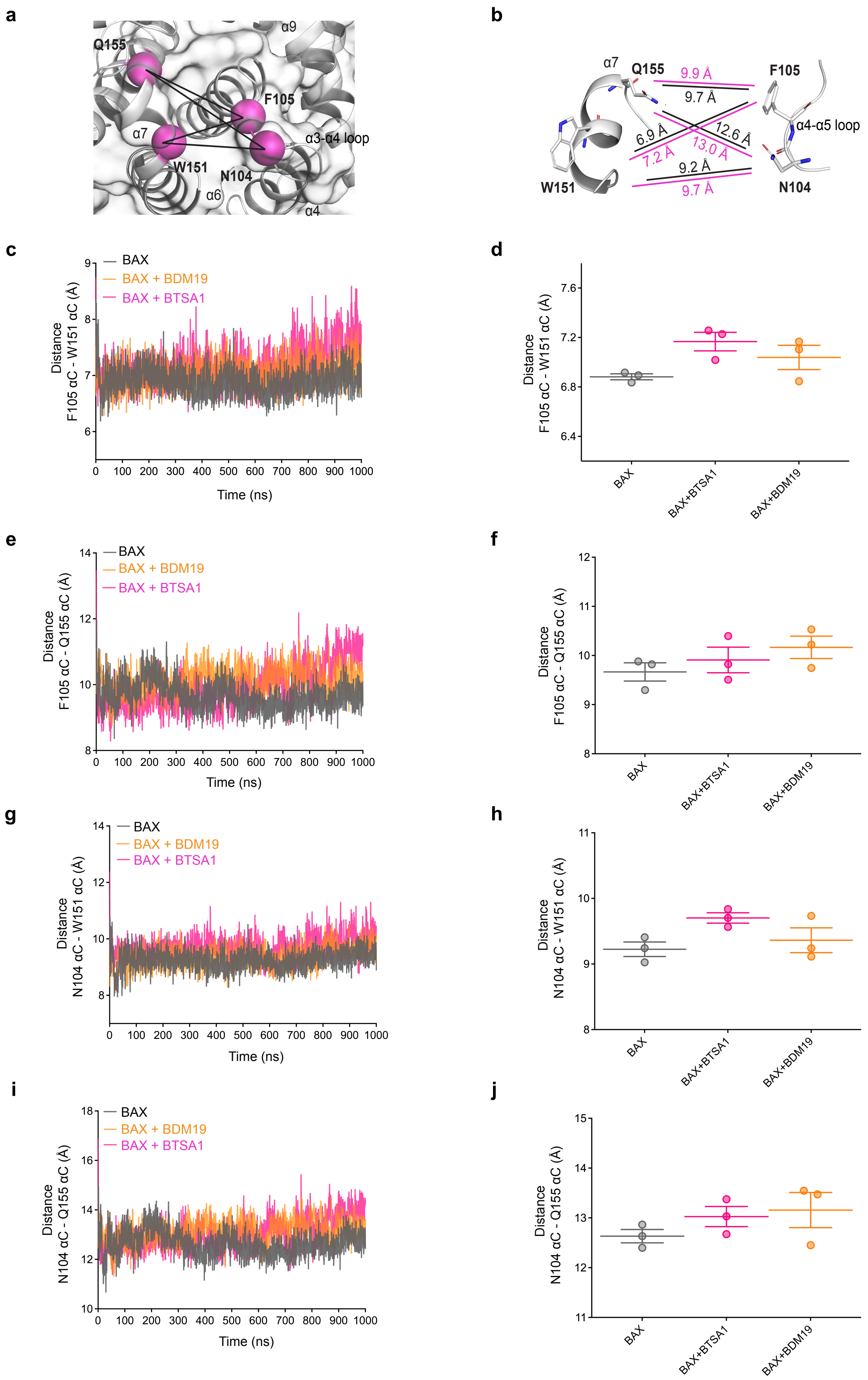
a
Q155
α9
F105
α7
α3-α4 loop
W151
N104
α6
α4
b
α7
Q155
9.9 Å
F105
9.7 Å
12.6 Å
α4-α5 loop
6.9 Å
13.0 Å
7.2 Å
9.2 Å
W151
9.7 Å
N104
c
BAX
BAX + BDM19
BAX + BTSA1
Distance
F105 αC - W151 αC (Å)
Time (ns)
d
Distance
F105 αC - W151 αC (Å)
BAX
BAX+BTSA1
BAX+BDM19
e
BAX
BAX + BDM19
BAX + BTSA1
Distance
F105 αC - Q155 αC (Å)
Time (ns)
f
Distance
F105 αC - Q155 αC (Å)
BAX
BAX+BTSA1
BAX+BDM19
g
BAX
BAX + BDM19
BAX + BTSA1
Distance
N104 αC - W151 αC (Å)
Time (ns)
h
Distance
N104 αC - W151 αC (Å)
BAX
BAX+BTSA1
BAX+BDM19
i
BAX
BAX + BDM19
BAX + BTSA1
Distance
N104 αC - Q155 αC (Å)
Time (ns)
j
Distance
N104 αC - Q155 αC (Å)
BAX
BAX+BTSA1
BAX+BDM19

Supplementary Fig. 9. Distance analysis of unbound BAX, the BAX-BDM19 and the BAX-BTSA1 complex molecular dynamics (MD) simulations in the α7/α4-α5 loop interface.

a-b Changes in structure and dynamics of the α7/α4-α5 loop interface in the BAX-BTSA1 complex. Transparent surface with ribbon representation of α7/α4-α5 loop interface with main residues highlighted in magenta (left), and graphical representation of distances between residues at the α7/α4-α5 loop interface (right). **c-j** Molecular dynamic simulation distances between (**c, d**) F105 and W151, (**e, f**) F105 and Q155, (**g, h**) N105 and W151, and (**i, j**) N104 and Q155 of unbound BAX, BAX-BDM19 and BAX-BTSA1. Distances are calculated from α-carbons and represented as distance with respect to time (**c**, **e**, **g**, **i**) or the average of all distances over 1000 ns (**d**, **f**, **h**, **j**) . All data represent mean n=3 simulations for unbound BAX, BAX-BDM19 and BAX-BTSA1 complex MD simulations. (**d**, **f**, **h**, **j**) Data are mean ± SEM from n=3 independent simulations. Source data are provided.

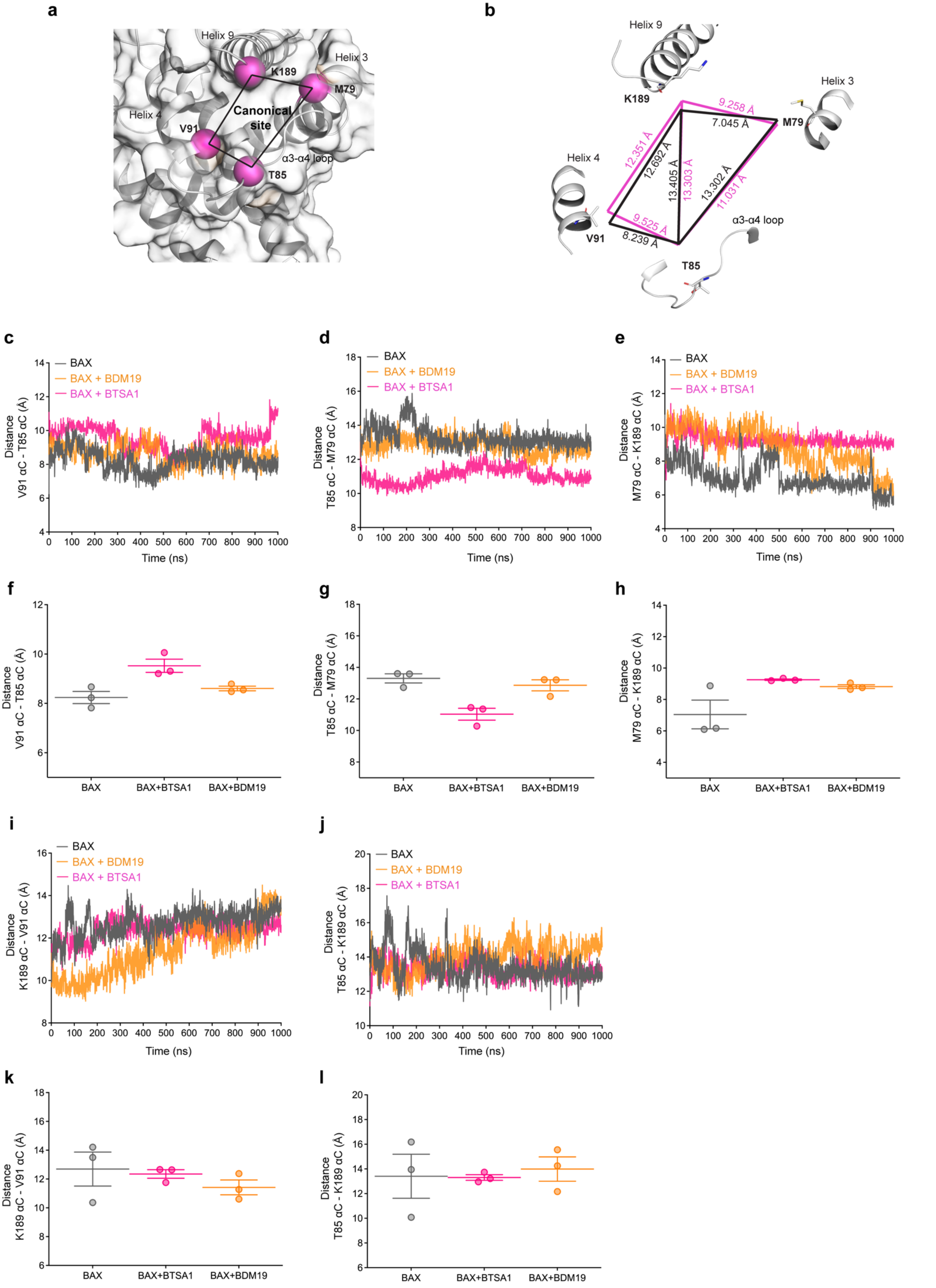
a
Helix 9
Helix 3
K189
M79
Helix 4
Canonical site
V91
α3-α4 loop
T85
b
Helix 9
K189
Helix 3
9.258 Å
7.045 Å
M79
12.351 Å
12.692 Å
13.405 Å
13.303 Å
13.302 Å
11.031 Å
Helix 4
9.525 Å
8.239 Å
V91
α3-α4 loop
T85
c
BAX
BAX + BDM19
BAX + BTSA1
Distance V91 αC - T85 αC (Å)
Time (ns)
d
Distance T85 αC - M79 αC (Å)
e
Distance M79 αC - K189 αC (Å)
f
g
h
BAX
BAX+BTSA1
BAX+BDM19
i
Distance K189 αC - V91 αC (Å)
j
Distance T85 αC - K189 αC (Å)
k
l

Supplementary Fig. 10. Distance analysis of unbound BAX, the BAX-BDM19 and the BAX-BTSA1 complex molecular dynamics (MD) simulations in the canonical site.

a-b Changes in structure and dynamics of the canonical site opening formed by α3, α3-α4 loop, α4, and α9 in the BAX-BTSA1 complex. Transparent surface with ribbon representation of the canonical site opening with main residues highlighted in magenta (left), and graphical representation of distances between residues at the canonical site opening (right). **c-l** Molecular dynamic simulation distances between (**c, f**) V91 and T85, (**d, g**) T85 and M79, (**e, h**) M79 and K189, (**i, k**) K189 and V91, and (**j, l**) T85 and K189. Distances are calculated from α-carbons and represented as distance with respect to time (**c-e**, **i-j**) or average of all distances over 1000 ns (**f-h**, **k-l**). All data represent mean n=3 simulations for both BAX, BAX-BDM19 and BAX-BTSA1 MD simulations. (**f-h**, **k-l**) Data are mean ± SEM from n=3 independent simulations. Source data are provided.

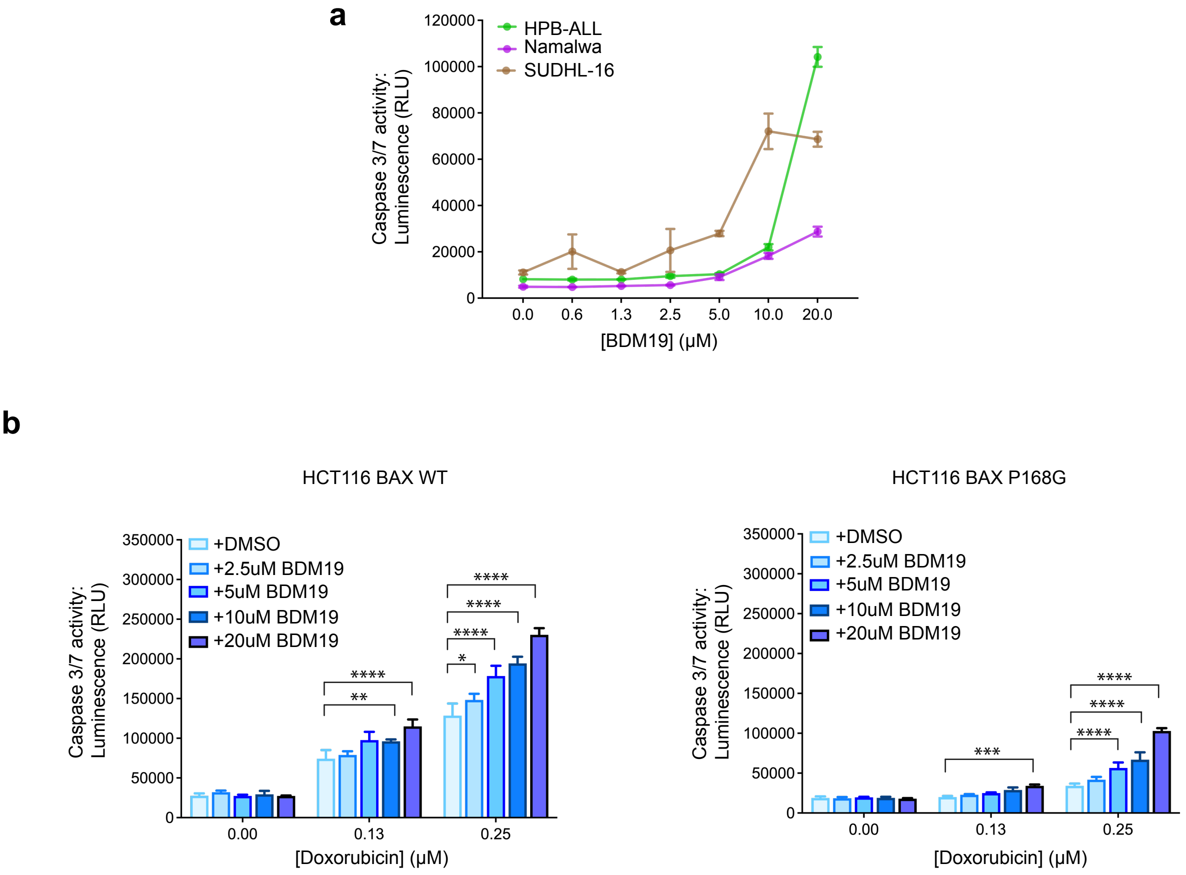

Supplementary Fig. 11. BDM19 promotes caspase 3/7 activity as single agent treatment in hematological cell lines and sensitizes cells expressing the BAX P168G resistant mutant.

a Caspase 3/7 activity of hematological HPB-ALL, Namalwa and SUDHL-16 cell lines treated with various concentrations of BDM19 for 6 hrs. Data are mean ± SEM and representative of n=2 independent experiments. **b** Caspase 3/7 activity of HCT116 BAX KO reconstituted with BAX WT (cytosolic dimer) or mutant BAX P168G (cytosolic dimer) treated with a titration of doxorubicin and fixed concentrations of BDM19 at 36hrs. Data are mean ± SEM and representative of n=3 independent experiments. Source data are provided.

Supplementary Table 1. Compounds selected from *in silico* screen and experimentally screened using BN-PAGE assay for disruption of cytosolic inactive BAX dimer and FPA assay for binding to recombinant BAX and competing FITC-BIM SAHB binding.

Compound	Catalog number	Chemical Structure	% Inactive BAX Dimer (BN-PAGE)	% FITC-BIM SAHB bound to BAX (FPA)
BDM1	16443695	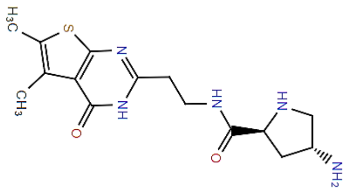	93	92
BDM2	44444006	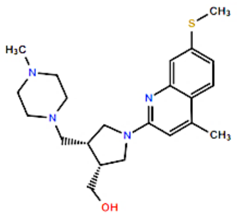	88	75
BDM3	15752051	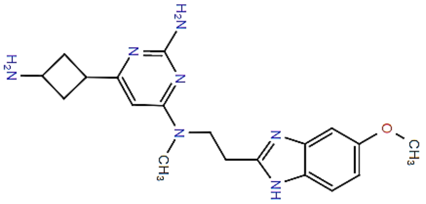	90	46
BDM4	49845931	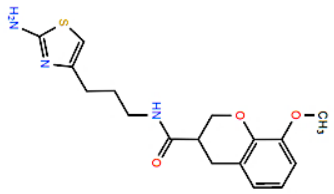	80	91
BDM5	6049747	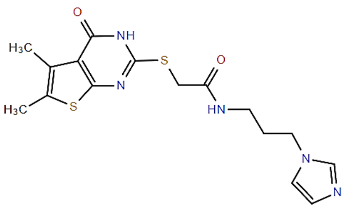	91	99

BDM6	E713-0165F	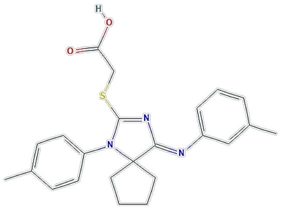	96	90
BDM7	5750-2696	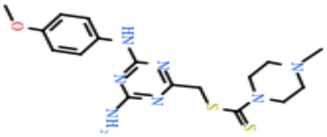	95	96
BDM8	M503-1887	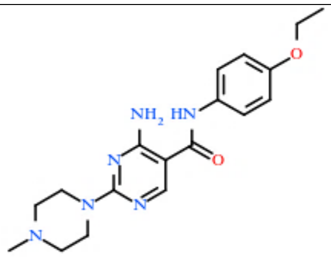	93	58
BDM9	S824-2057	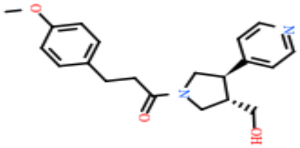	106	126
BDM10	F085-0940	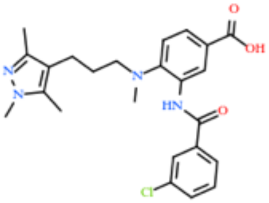	81	35
BDM11	8020-4552	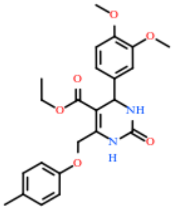	72	99
BDM12	3740-0905	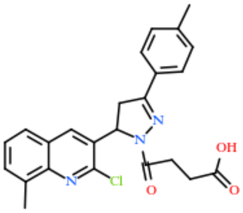	81	76

BDM13	F085-0884	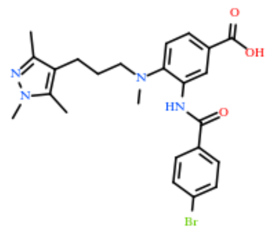	90	66
BDM14	8010-6301	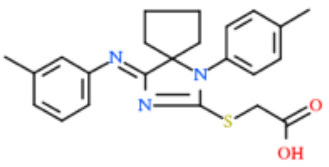	76	95
BDM15	V027-2558	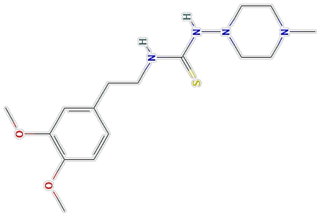	73	74
BDM16	STL369066	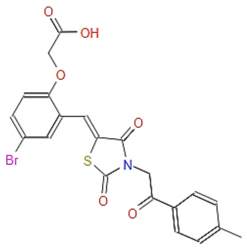	61	32
BDM17	STK190287	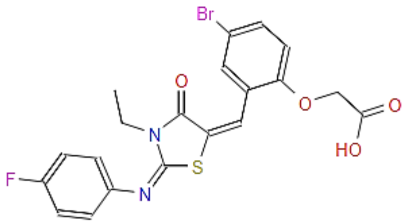	36	6
BDM18	STK592161	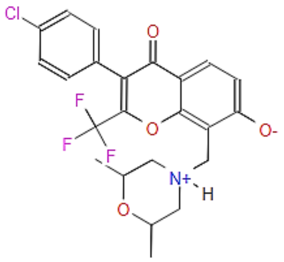	77	99
BDM19	STK995952		63	< 0

		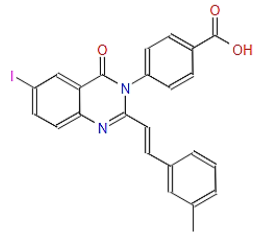		
BDM20	STK653270	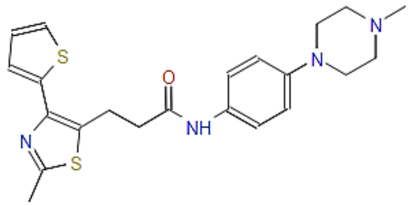	92	142
BDM21	STK601803	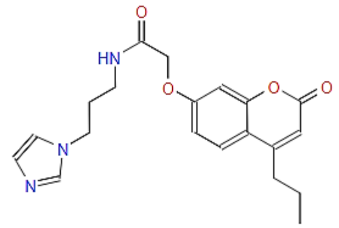	78	108
BDM22	STK458371	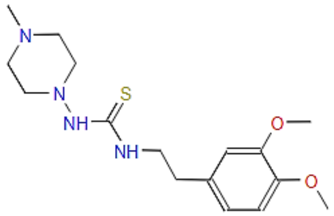	101	116
BDM23	Z96732856	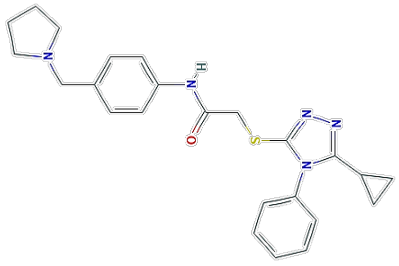	109	197
BDM24	Z1019703536	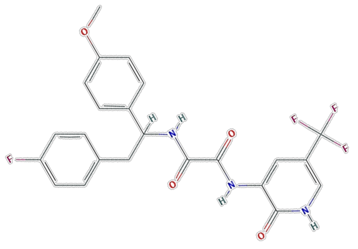	78	208

BDM25	Z1450241095	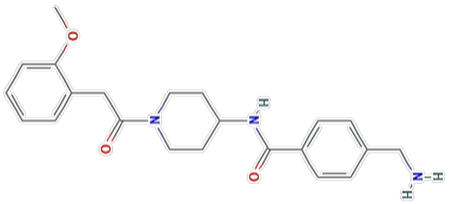	65	103
BDM26	Z2074706602	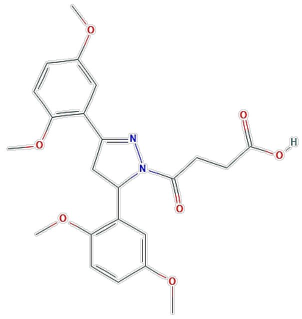	89	100

Supplementary Methods

Chemical Synthesis of *(E)-4-(6-iodo-2-(3-methylstyryl)-4-oxoquinazolin-3(4H)-yl)benzoic acid* (BDM19)

Scheme of Synthesis

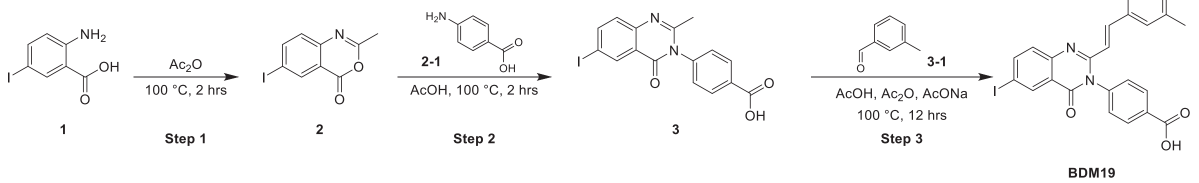

Step 1: General procedure for preparation of compound 2

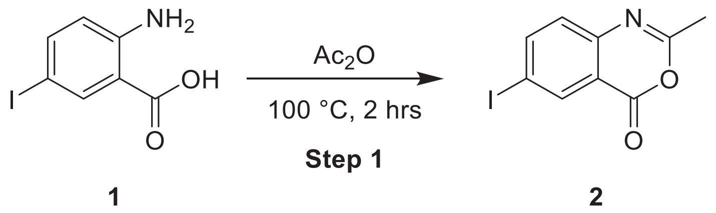

To a solution of compound **1** (10.0 g, 38.0 mmol, 1.00 eq) in Ac_2O (150 mL) was stirred at 100 °C for 2 hrs. LCMS (RT = 0.639 min) showed compound **1** was consumed completely and 86.9% of compound **2** was detected. The reaction mixture was cooled to 0 °C, filtered and washed by *n*-hexane (50.0 mL). The solid was collected and concentrated under reduced pressure to give compound **2** (11.0 g, crude) as a white solid.

LCMS: RT = 0.639 min, m/z = 288.1 $[M+H]^+$

Step 2: General procedure for preparation of compound 3

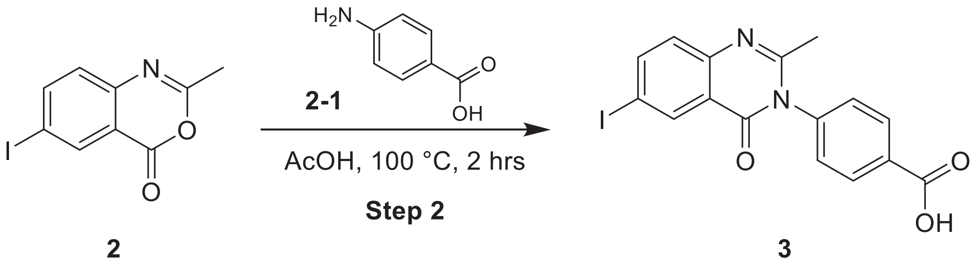

To a solution of compound **2** (10.0 g, 34.8 mmol, 1.00 eq) in AcOH (100 mL) was added compound **2-1** (5.73 g, 41.8 mmol, 1.20 eq) at 25 °C. The mixture was stirred at 100 °C for 2 hrs. LCMS (RT = 0.487 min) showed compound **2** was consumed completely and 57.4% of compound **3** was detected. The reaction mixture was cooled to 0 °C, filtered and washed by *n*-hexane (50.0 mL). The solid was

collected and was purified by prep-HPLC (neutral condition) to give compound **3** (4.00 g, 8.37 mmol, 24.0% yield) as a yellow solid.

LCMS: RT = 0.487 min, m/z = 407.1 $[M+H]^+$

Step 3: General procedure for preparation of compound BDM19

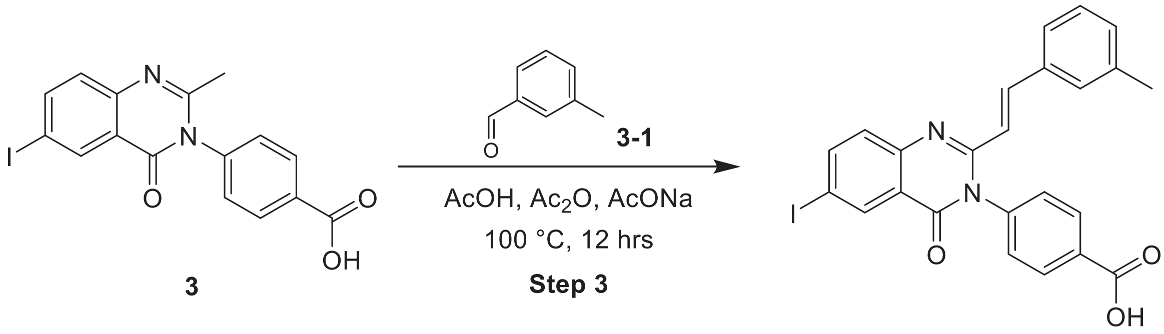

BDM19

To a solution of compound **3** (800 mg, 1.97 mmol, 1.00 eq) in Ac_2O (5.00 mL) and AcOH (10.0 mL) was added compound **3-1** (710 mg, 5.91 mmol, 3.00 eq) and AcONa (808 mg, 9.85 mmol, 5.00 eq). The mixture was stirred at 100 °C for 12 hrs. LCMS (RT=0.560 min) showed 40.6% of compound **3** was remained and 34.5% of **BDM19** was detected. The reaction mixture was diluted with H_2O 50.0 mL and extracted with EtOAc 60.0 mL (30.0 mL * 2). The organic phase was washed with brine 40.0 mL, dried over Na_2SO_4, filtered and concentrated under reduced pressure to give a residue. The crude product was purified by reversed-phase HPLC (neutral condition) to give **BDM19** (100 mg, 126 μmol, 98.5% purity, 11.1% yield) as a yellow solid.

LCMS: RT = 2.048 mins, m/z = 509.1 $[M+H]^+$

HPLC: RT = 2.013 mins, 98.5% purity

^1H NMR: (400 MHz, DMSO-*d6*)

δ 8.41 - 8.38 (m, 1H), 8.18 - 8.14 (m, 1H), 8.13 - 8.09 (m, 2H), 7.89 - 7.83 (m, 1H), 7.58 - 7.54 (m, 1H), 7.53 - 7.49 (m, 2H), 7.27 - 7.18 (m, 2H), 7.18 - 7.11 (m, 2H), 6.31 - 6.25 (m, 1H), 2.26 (s, 3H)

Chemical Synthesis of *(E)-4-(2-(2-cyclopentylvinyl)-6-iodo-4-oxoquinazolin-3(4H)-yl)benzoic acid* (BDM19.2)

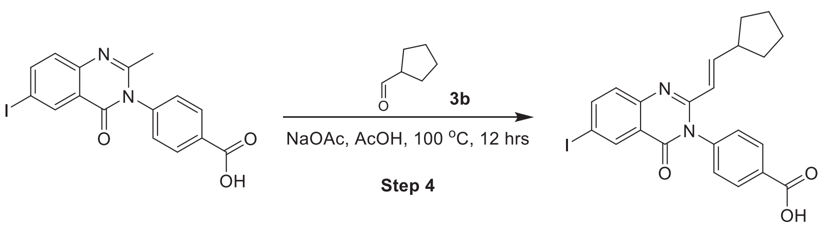

3 BDM19.2

Step 4: General procedure for preparation of compound BDM19.2

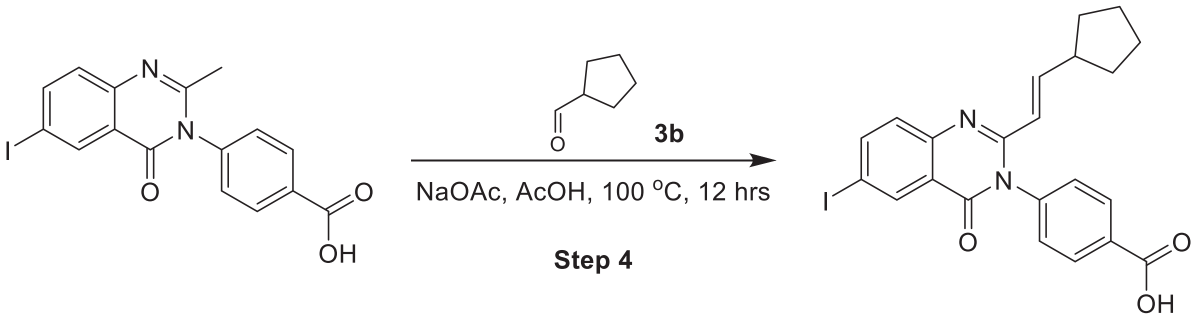

3 **BDM19.2**

To a solution of compound **3** (3.00 g, 7.39 mmol, 1.00 eq) in AcOH (30.0 mL) was added NaOAc (18.2 g, 221 mmol, 30.0 eq) and compound **3b** (1.09 g, 11.1 mmol, 1.50 eq) at 25 °C. The mixture was stirred at 100 °C for 12 hrs. TLC (Petroleum ether : EtOAc = 2 : 1, compound **3** R_f = 0.5, **BDM19.2** R_f = 0.6) indicated ~50% of compound **3** was remained, and one major new spot with lower polarity was detected. The reaction mixture was diluted with H_2O 50.0 mL and extracted with EtOAc 60.0 mL (30.0 mL * 2). The organic phase was washed with brine 40.0 mL, dried over Na_2SO_4, filtered and concentrated under reduced pressure to give a residue. The crude product was purified by reversed-phase HPLC (neutral condition) to give **BDM19.2** (35.0 mg, 67.4 μmol, 93.7% purity, 3.70% yield) as a yellow solid.

LCMS: RT = 2.087 mins, m/z = 486.9 $[M+H]^+$

HPLC: RT = 2.049 mins, 93.7% purity

^{1}H NMR: (400 MHz, $CDCl_3$)

δ 8.59 (d, *J* = 1.4 Hz, 1H), 8.29 (d, *J* = 8.1 Hz, 2H), 8.04 (d, *J* = 2.1, 8.7 Hz, 1H), 7.48 (d, *J* = 8.6 Hz, 1H), 7.38 (d, *J* = 8.4 Hz, 2H), 7.20 (d, *J* = 8.4, 15.0 Hz, 1H), 5.67 (d, *J* = 15.1 Hz, 1H), 2.47 (d, *J* = 7.6 Hz, 1H), 1.78 - 1.73 (m, 2H), 1.68 - 1.63 (m, 2H), 1.59 - 1.53 (m, 2H), 1.40 - 1.32 (m, 2H)

Chemical Synthesis of *(E)-4-(2-(2-cyclopropylvinyl)-6-iodo-4-oxoquinazolin-3(4H)-yl)benzoic acid* (BDM19.3)

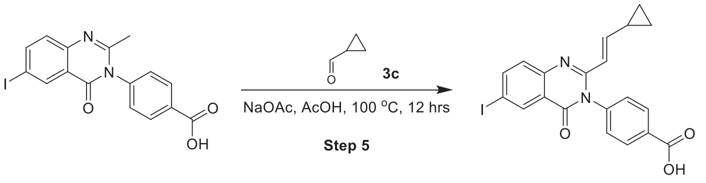

3 BDM19.3

Step 5: General procedure for preparation of compound BDM19.3

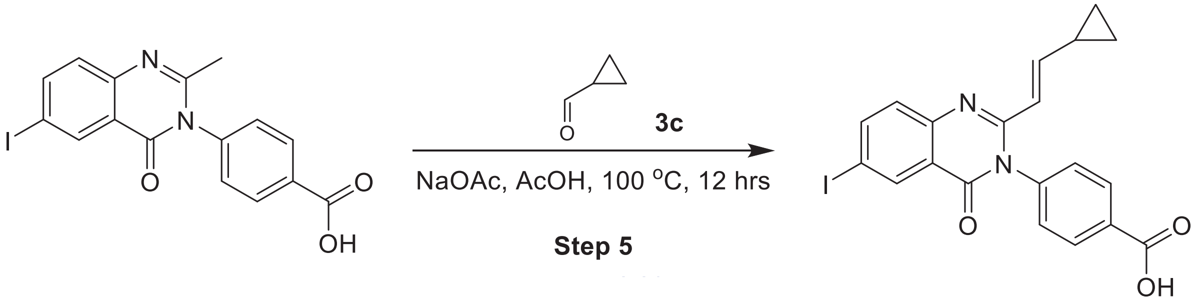

3 BDM19.3

To a solution of compound **3** (800 mg, 1.97 mmol, 1.00 eq) in AcOH (15.0 mL) was added compound **3c** (207 mg, 2.95 mmol, 1.50 eq) and AcONa (4.85 g, 59.1 mmol, 30.0 eq). The mixture was stirred at 80 °C for 12 hrs. LCMS (RT = 0.569 min, SM: RT = 0.492) showed 67.3% of compound **3** was remained and 24.1% of **BDM19.3** was detected. The reaction mixture was diluted with H_2O 50.0 mL and extracted with EtOAc 60.0 mL (30.0 mL * 2). The organic phase was washed with brine 40.0 mL, dried over Na_2SO_4, filtered and concentrated under reduced pressure to give a residue. The crude product was purified by reversed-phase HPLC (neutral condition) to give **BDM19.3** (60.0 mg, 131 µmol, 15.0% yield) as a yellow solid.

LCMS: RT = 1.793 min, m/z = 459.3 [M+H]

HPLC: RT = 1.688 mins, 97.3% purity

^{1}H NMR: (400 MHz, DMSO-*d6*) δ 8.35 - 8.31 (m, 1H), 8.16 - 8.04 (m, 3H), 7.52 - 7.40 (m, 3H), 6.67 - 6.57 (m, 1H), 5.76 - 5.67 (m, 1H), 1.56 - 1.44 (m, 1H), 0.89 - 0.80 (m, 2H), 0.68 - 0.57 (m, 2H)

Chemical Synthesis of *(E)-4-(2-(3-methylstyryl)-4-oxo-5,6,7,8-tetrahydroquinazolin-3(4H)-yl)benzoic acid* (BDM19.4)

<u>Scheme of Synthesis</u>

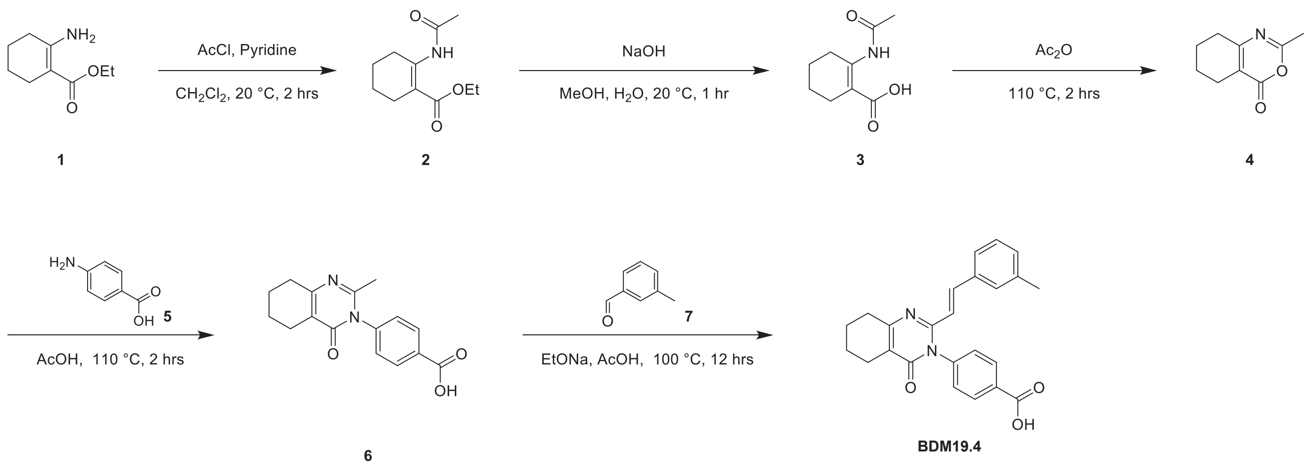

Step 1: General procedure for preparation of compound 2

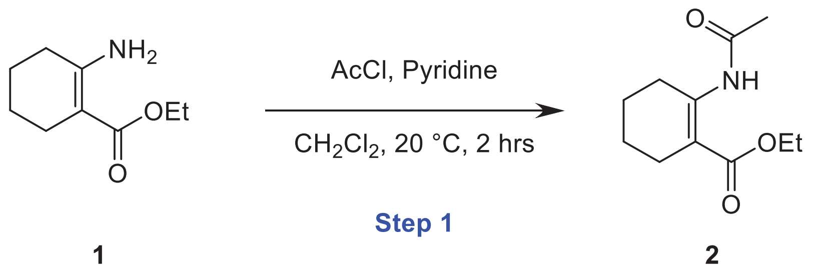

To a solution of compound **1** (5.00 g, 29.6 mmol, 1.00 *eq*) in CH_2Cl_2 (100 mL) was added Pyridine (935 mg, 11.8 mmol, 954 µL, 0.40 *eq*) and acetyl chloride (11.6 g, 148 mmol, 10.5 mL, 5.00 *eq*). The mixture was stirred at 20 °C for 2 hrs. TLC (Petroleum ether : Ethyl acetate = 5 : 1, compound **1** R_f = 0.10, compound **2** R_f = 0.40) indicated compound **1** was consumed completely. The reaction mixture was quenched by MeOH (50.0 mL) at 0 °C, and then filtered and concentrated under reduced pressure to give compound **2** (3.60 g, 17.0 mmol, 57.7% yield) as a yellow solid.

Step 2: General procedure for preparation of compound 3

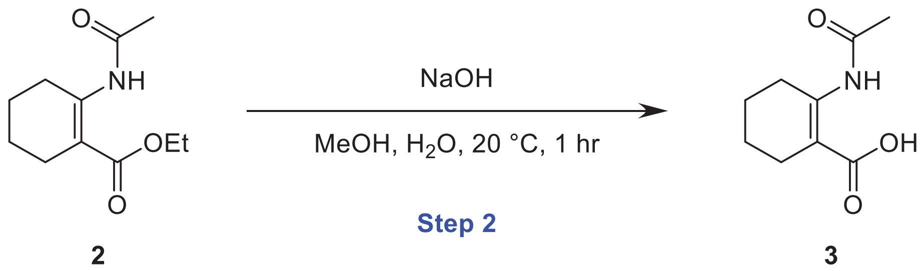

A mixture of compound **2** (3.60 g, 17.0 mmol, 1.00 *eq*) and NaOH (3.41 g, 85.2 mmol, 5.00 eq) in MeOH (50.0 mL) and H_2O (10.0 mL) was degassed and purged with N_2 for 3 times, and then the mixture was stirred at 20 °C for 1 hr under N_2 atmosphere. TLC (Petroleum ether : Ethyl acetate = 5 : 1, compound **2** R_f = 0.40, compound **3** R_f = 0.10) indicated compound **2** was consumed completely. The reaction

mixture was filtered and concentrated under reduced pressure to give compound **3** (2.98 g, 16.3 mmol, 95.5% yield) as a white solid.

^{1}H NMR: EC18355-14-P3 (400 MHz, CHLOROFORM-*d*) δ 11.42 (s, 1H), 3.04 - 2.93 (m, 2H), 2.40 - 2.31 (m, 2H), 2.11 (s, 3H), 1.72 - 1.53 (m, 5H).

Step 3: General procedure for preparation of compound 4

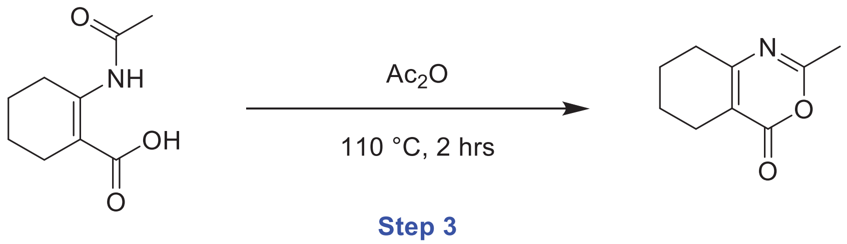

3 **4**

A mixture of compound **3** (2.00 g, 10.9 mmol, 1.00 *eq*) in Ac_2O (20.0 mL) was degassed and purged with N_2 for 3 times, and then the mixture was stirred at 110 °C for 2 hrs under N_2 atmosphere. TLC (Petroleum ether : Ethyl acetate = 3 : 1, compound **3** R_f = 0.20, compound **4** R_f = 0.40) indicated compound **3** was consumed completely. The reaction mixture was quenched by H_2O (20.0 mL) and extracted with EtOAc (20.0 mL * 3). The combined organic layers were washed with brine (15.0 mL * 2), dried over anhydrous Na_2SO_4, filtered and concentrated under reduced pressure to give compound **4** (1.80 g, crude) as a yellow solid.

Step 4: General procedure for preparation of compound 6

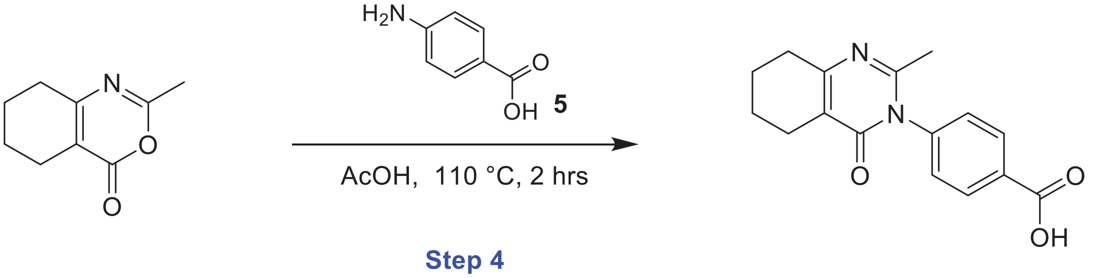

4 **6**

A mixture of compound **4** (1.80 g, 10.9 mmol, 1.00 *eq*), compound **5** (1.79 g, 13.1 mmol, 1.20 *eq*) in AcOH (20.0 mL) was degassed and purged with N_2 for 3 times, and then the mixture was stirred at 110 °C for 2 hrs under N_2 atmosphere. LCMS showed compound **4** was consumed completely. The reaction mixture was filtered and concentrated under reduced pressure to give a residue. The residue was purified by prep-HPLC (TFA condition) to give compound **5** (330 mg, 1.16 mmol, 10.6% yield) as a yellow solid.

LCMS: RT = 0.276 min, m/z+1 = 285.2 $[M+H]^+$

Step 5: General procedure for preparation of BDM19.4

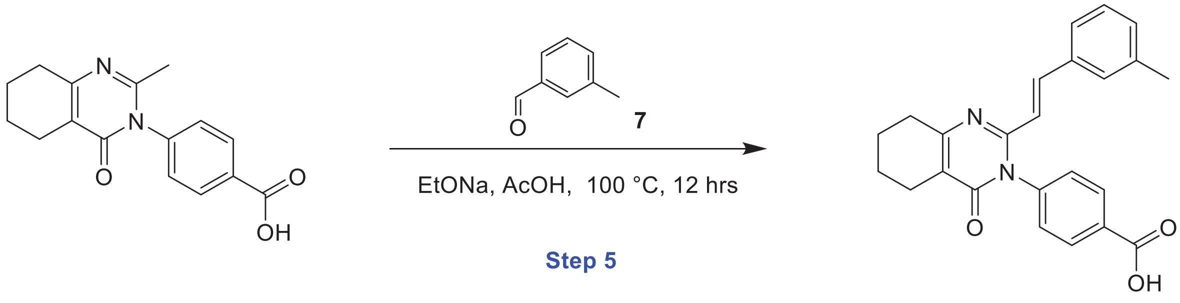

6 BDM19.4

A mixture of compound **6** (300 mg, 1.05 mmol, 1.00 *eq*), compound **7** (380 mg, 3.17 mmol, 3.00 *eq*), NaOAc (433 mg, 5.30 mmol, 5.00 *eq*) in AcOH (10.0 mL) was degassed and purged with N_2 for 3 times, and then the mixture was stirred at 100 °C for 12 hrs under N_2 atmosphere. LCMS showed 2.93% of compound **6** was remained. The reaction mixture was filtered and concentrated under reduced pressure to give a residue. The crude product was purified by prep-TLC (Petroleum ether : Ethyl acetate = 0 : 1, **BDM19.4** : R_f = 0.40) to give **BDM19.4** (10.0 mg, 24.8 μmol, 3.53% yield, 96.3% purity) as a white solid.

LCMS: RT = 2.084 min, m/z+1 = 387.1 $[M+H]^+$

HPLC: RT = 1.373 min, purity: 98.3%

^{1}H NMR: (400 MHz, METHANOL-*d4*) δ 8.23 (d, J = 5.3 Hz, 2H), 7.77 (d, *J* = 15.6 Hz, 1H), 7.41 - 7.36 (m, 2H), 7.21 - 7.03 (m, 4H), 6.28 (d, *J* = 15.5 Hz, 1H), 2.75 (t, *J* = 5.8 Hz, 2H), 2.52 (t, *J* = 5.9 Hz, 2H), 2.27 (s, 3H), 1.93 - 1.78 (m, 4H).

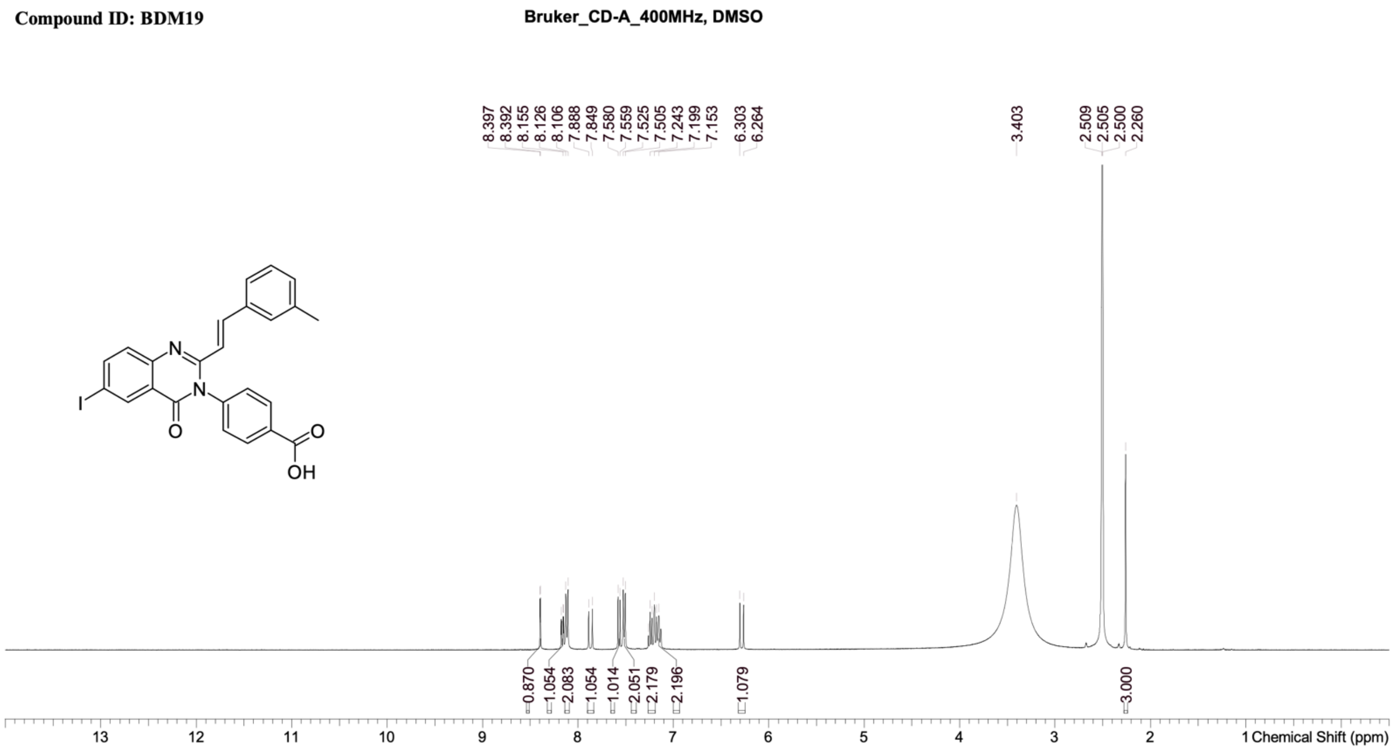

Supplementary Fig. 12. ^{1}H NMR of BDM19

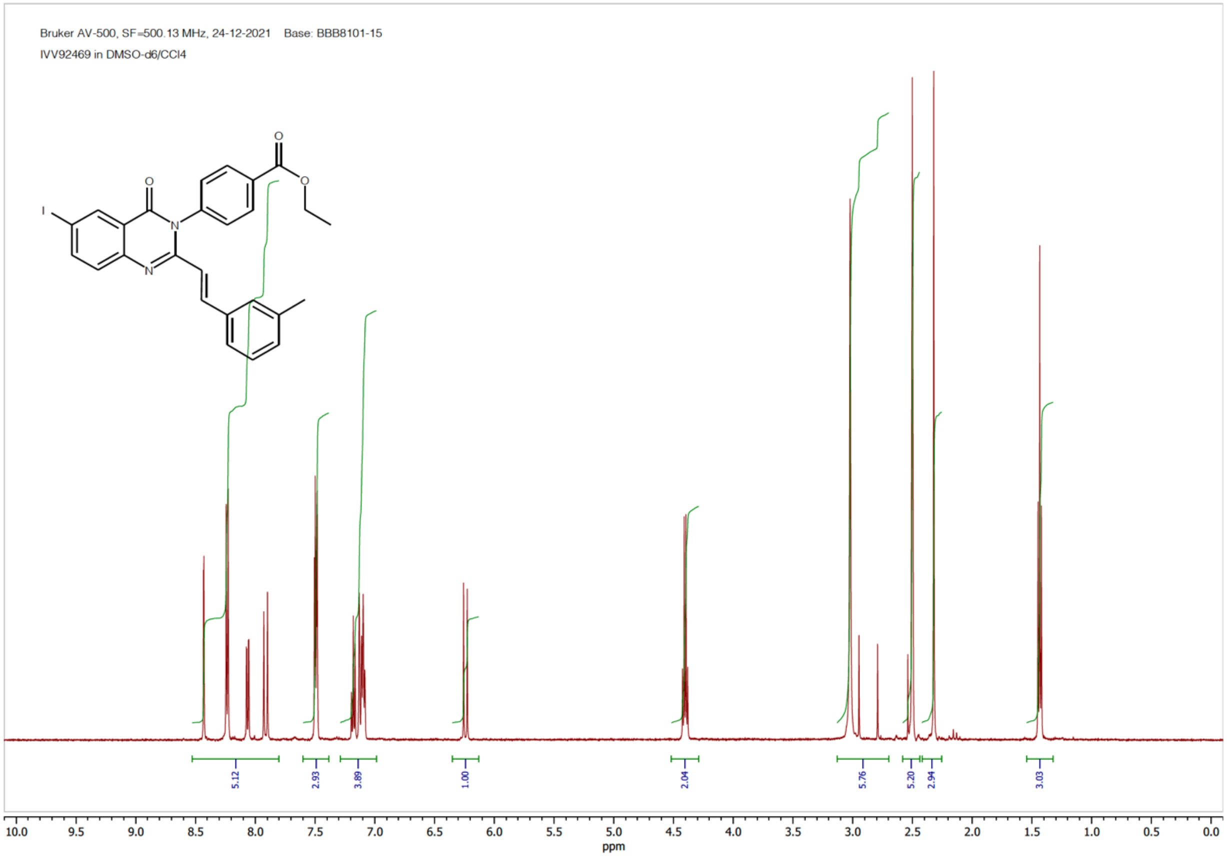

Supplementary Fig. 13. ^{1}H NMR of BDM19.1

Compound ID: BDM19.2

Bruker_CD-C_400MHz, CDCl3

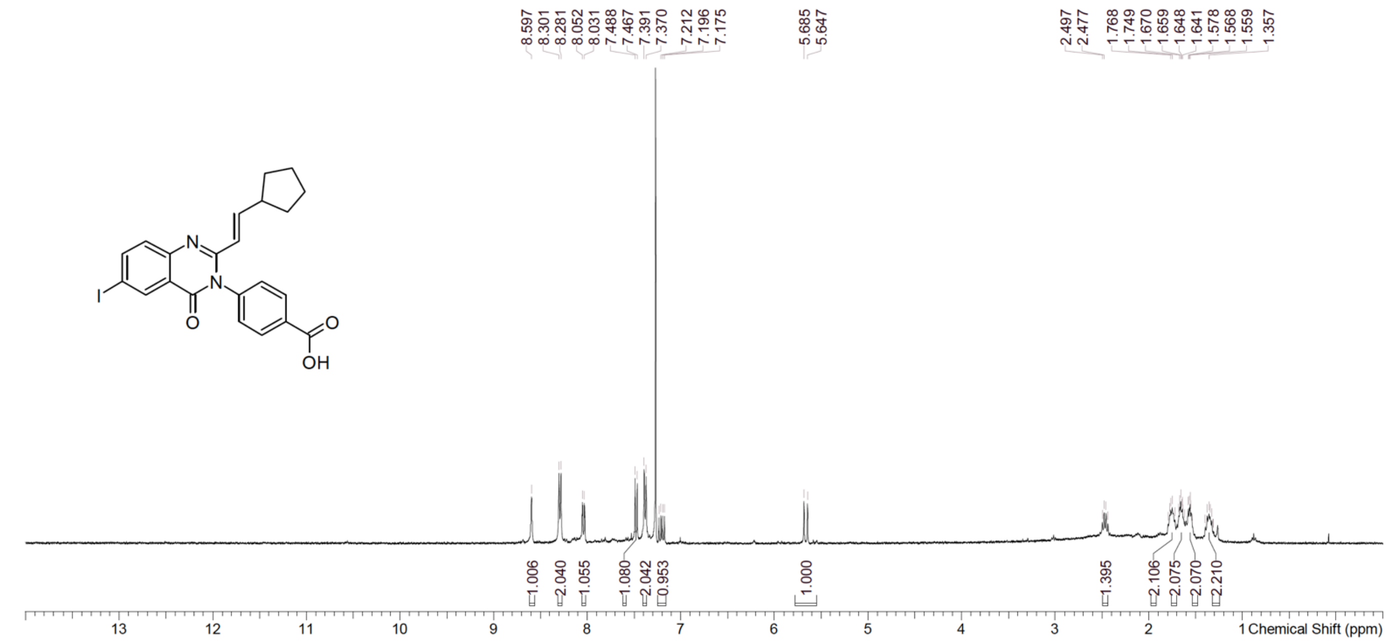

Supplementary Fig. 14. ^{1}H NMR of BDM19.2

Compound ID: BDM19.3

Bruker_CD-B_400MHz, DMSO

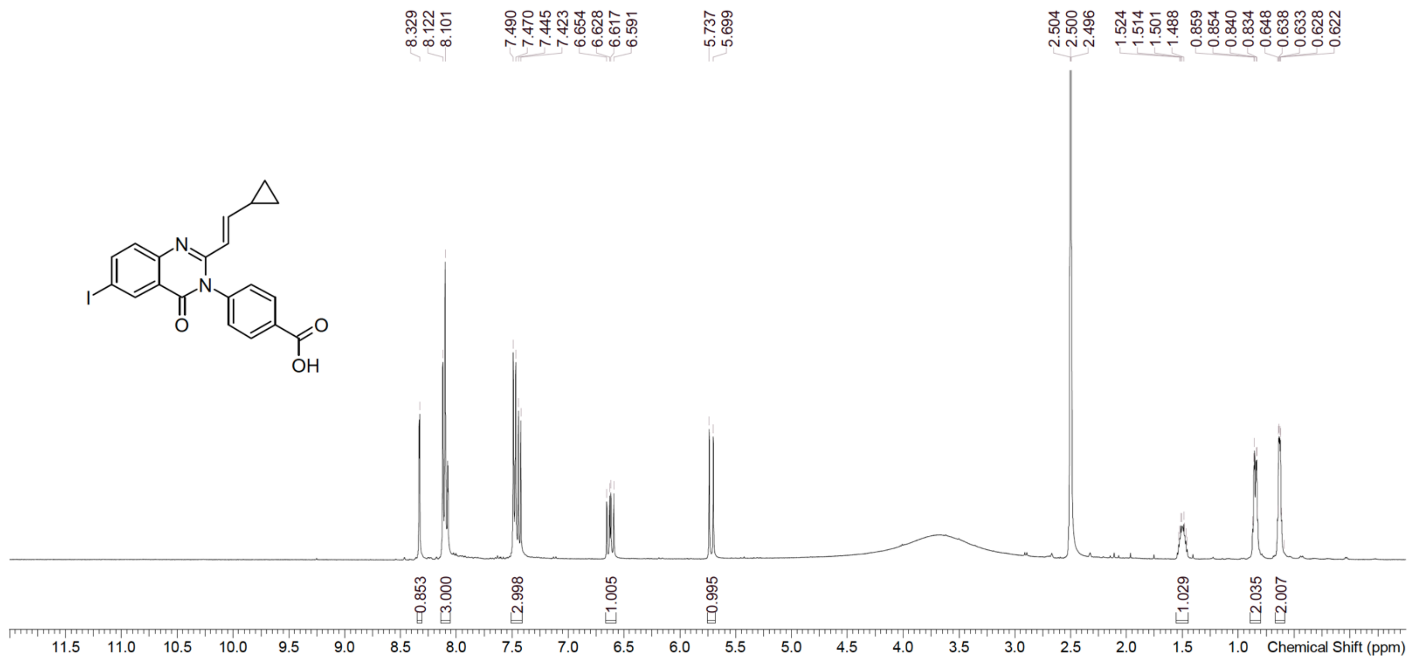

Supplementary Fig. 15. ^{1}H NMR of BDM19.3

Compound ID: BDM19.4

Bruker_CD-A_400MHz, MeoD

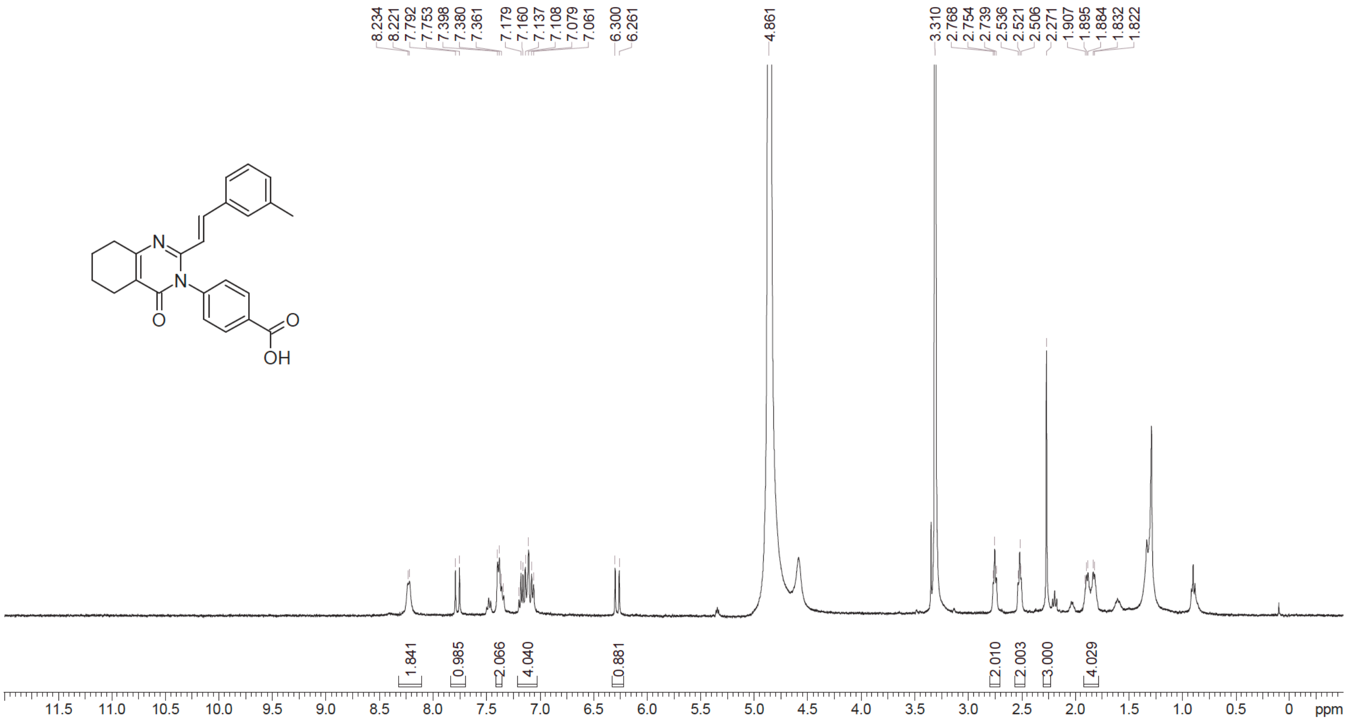

Supplementary Fig. 16. ^{1}H NMR of BDM19.4
