# Supplementary material for: Chemical modulation of cytosolic BAX homodimer potentiates BAX activation and apoptosis
Source: Nat Commun. 2023 Dec 16;14:8381. doi: 10.1038/s41467-023-44084-3 (PMC10725471; doi:10.1038/s41467-023-44084-3)
Supplement: Supplementary file 3 — Reporting Summary [file 41467_2023_44084_MOESM3_ESM.pdf]

nature portfolio

Corresponding author(s): Evripidis Gavathiotis

Last updated by author(s): November 10, 2023

Reporting Summary

Nature Portfolio wishes to improve the reproducibility of the work that we publish. This form provides structure for consistency and transparency in reporting. For further information on Nature Portfolio policies, see our Editorial Policies and the Editorial Policy Checklist.

Statistics

For all statistical analyses, confirm that the following items are present in the figure legend, table legend, main text, or Methods section.

n/a	Confirmed	
☐	☒	The exact sample size (*n*) for each experimental group/condition, given as a discrete number and unit of measurement
☐	☒	A statement on whether measurements were taken from distinct samples or whether the same sample was measured repeatedly
☐	☒	The statistical test(s) used AND whether they are one- or two-sided<br>*Only common tests should be described solely by name; describe more complex techniques in the Methods section.*
☐	☒	A description of all covariates tested
☐	☒	A description of any assumptions or corrections, such as tests of normality and adjustment for multiple comparisons
☐	☒	A full description of the statistical parameters including central tendency (e.g. means) or other basic estimates (e.g. regression coefficient) AND variation (e.g. standard deviation) or associated estimates of uncertainty (e.g. confidence intervals)
☐	☒	For null hypothesis testing, the test statistic (e.g. *F*, *t*, *r*) with confidence intervals, effect sizes, degrees of freedom and *P* value noted<br>*Give P values as exact values whenever suitable.*
☒	☐	For Bayesian analysis, information on the choice of priors and Markov chain Monte Carlo settings
☒	☐	For hierarchical and complex designs, identification of the appropriate level for tests and full reporting of outcomes
☒	☐	Estimates of effect sizes (e.g. Cohen's *d*, Pearson's *r*), indicating how they were calculated

Our web collection on statistics for biologists contains articles on many of the points above.

Software and code

Policy information about availability of computer code

Data collection	Western blot data were collected with Odyssey Infrared Imaging System, Image Studio (Version 3.1, LI-COR Biosciences) or x-ray film for chemiluminescence. Modeling data were collected and analyzed with Schrodinger software suite (Releases 2018-2021, c, LLC) and Pymol (Version 2.3-2.4, The PyMOL Molecular Graphics System, Schrodinger, LLC). NMR data were collected and processed with TopSpin 3.6.2 software. MST Data was acquired with NanoTemper MO.Control 2 (2019, NanoTemper).
Data analysis	Data analysis and statistical comparisons were performed by Graphpad Prism 8.0-9.0 software. Western blot data were analyzed with Image Studio (Version 3.1, LICOR). The BLISS calculations were determined using the Combenefit program, version (2.021). NMR data were analyzed with CcpNmr Analysis 2.5.2. Structural and small molecule data were analyzed with modules PHASE, CANVAS, GLIDE, EPIC, LIGPREP, MAESTRO, DESMOND, and IFD using Schrodinger Software Suite version 2018-2021. MST Data was analyzed using Nano Temper MO.Affinity Analysis 3 (2019, NanoTemper).

For manuscripts utilizing custom algorithms or software that are central to the research but not yet described in published literature, software must be made available to editors and reviewers. We strongly encourage code deposition in a community repository (e.g. GitHub). See the Nature Portfolio guidelines for submitting code & software for further information.

Data

Policy information about availability of data

All manuscripts must include a data availability statement. This statement should provide the following information, where applicable:

- Accession codes, unique identifiers, or web links for publicly available datasets
- A description of any restrictions on data availability
- For clinical datasets or third party data, please ensure that the statement adheres to our policy

All data generated or analyzed during this study are included in this published article and its supplementary information files. Source data are provided with this paper. The following publicly available data sets were used in the production of this manuscript:
PDB 1F16 doi: 10.2210/pdb1F16/pdb
PDB 4S0O doi: 10.2210/pdb4S0O/pdb

Research involving human participants, their data, or biological material

Policy information about studies with human participants or human data. See also policy information about sex, gender (identity/presentation), and sexual orientation and race, ethnicity and racism.

Reporting on sex and gender	N/A
Reporting on race, ethnicity, or other socially relevant groupings	N/A
Population characteristics	N/A
Recruitment	N/A
Ethics oversight	N/A

Note that full information on the approval of the study protocol must also be provided in the manuscript.

Field-specific reporting

Please select the one below that is the best fit for your research. If you are not sure, read the appropriate sections before making your selection.

☒ Life sciences ☐ Behavioural & social sciences ☐ Ecological, evolutionary & environmental sciences

For a reference copy of the document with all sections, see nature.com/documents/nr-reporting-summary-flat.pdf

Life sciences study design

All studies must disclose on these points even when the disclosure is negative.

Sample size	Sample sizes were designed based on related assays with our previous publications Garner et al. Molecular Cell 2016, 63:485-497, doi: 10.1016/j.molcel.2016.10.005; Reyna et al. Cancer Cell 2017, 32:490-505, doi: 10.1016/j.ccell.2017.09.001; Spitz et al. Nature Communications 2021, 12:1134, doi: 10.1038/s41467-021-21224-1 and Lopez et al. Nature Communications 2022, 13:1199, doi: 10.1038/s41467-022-28741-7. Sample sizes and statistical data are reported in figure legends.
Data exclusions	No data were excluded from the analyses.
Replication	Technical replicates and independent experiments were performed to verify reproducibility of the assays. All attempts at the replication were successful. The experimental findings were reliably reproduced as described in the figure legends.
Randomization	Allocation was random. For studies involving cells in culture, treatment groups were attributed randomly between wells and plates to account for well or plate positioning effects.
Blinding	Blinding was not required in this study as all comparisons were made using quantitative analysis of computational, biochemical or cellular data with no animal or human subjects and randomized group allocation was not performed.

Reporting for specific materials, systems and methods

We require information from authors about some types of materials, experimental systems and methods used in many studies. Here, indicate whether each material, system or method listed is relevant to your study. If you are not sure if a list item applies to your research, read the appropriate section before selecting a response.

Materials & experimental systems

n/a	Involved in the study
☐	☒ Antibodies
☐	☒ Eukaryotic cell lines
☒	☐ Palaeontology and archaeology
☒	☐ Animals and other organisms
☒	☐ Clinical data
☒	☐ Dual use research of concern
☒	☐ Plants

Methods

n/a	Involved in the study
☒	☐ ChIP-seq
☒	☐ Flow cytometry
☒	☐ MRI-based neuroimaging

Antibodies

Antibodies used

BCL-XL (Cell Signaling Cat. 2764). Dilution 1:1000
MCL-1 (Cell Signaling Cat. 5453). Dilution 1:1000
BAX (Cell signaling Cat. 2772). Dilution 1:1000
BAX (Santa Cruz Biotechnology Cat. sc-23959). Dilution 1:300
BCL-2 (Cell Signaling Cat. 4223). Dilution 1:1000
BAK (Cell Signaling Cat. 12105). Dilution 1:1000
BIM (Cell Signaling Cat. 2933). Dilution 1:1000
BID (Santa Cruz Biotechnology Cat. sc-11423). Dilution 1:500
Cleaved Caspase-3 (Cell Signaling Cat. 9664). Dilution 1:500
Cleaved PARP (Cell Signaling Cat. 5625). Dilution 1:1000
COX-IV (Cell Signaling Cat. 4850). Dilution 1:1000
β-actin (Sigma Cat. A1978). Dilution 1:10000
β-tubulin (Cell Signaling Cat. 2146). Dilution 1:1000
VDAC (Abcam Cat. 15895). Dilution 1:1000
Cytochrome c (BD Pharmigen Cat. 556433). Dilution 1:300
IRDye800CW Goat anti-rabbit (LICOR Cat. 926-32211). Dilution 1:10000
IRDye800CW Goat anti-mouse (LICOR Cat. 925-32210). Dilution 1:10000
IRDye680RD Goat anti-rabbit (LICOR Cat. 926-68071). Dilution 1:10000
IRDye680RD Goat anti-mouse (LICOR Cat. 926-68070). Dilution 1:10000
Anti-rabbit IgG HRP-linked (Cell Signaling Cat. 7074). Dilution 1:5000

Validation

All antibodies used in this study were from commercial sources and have been validated in the literature and our previous publications. Such information is provided in the manufacturer's website.
BCL-XL, Sample PMID: 36289220
MCL-1, Sample PMID: 35982046
BAX, Sample PMID: 33602934
BAX, Sample PMID: 29017059
BCL-2, Sample PMID: 36266274
BAK, Sample PMID: 35115713
BIM, Sample PMID: 33852868
BID, Sample PMID: 24735925
Cleaved Caspase-3, Sample PMID: 31453810
Cleaved PARP, Sample PMID: 33406417
COX-IV, Sample PMID:34556855
β-actin, Sample PMID: 36543799
β-tubulin, Sample PMID: 35933466
VDAC, Sample PMID: 31995728
Cytochrome c, Sample PMID: 34931711
IRDye800CW Goat anti-rabbit, Sample PMID: 31819006
IRDye800CW Goat anti-mouse, Sample PMID: 31819006
IRDye680RD Goat anti-rabbit, Sample PMID: 31819006
IRDye680RD Goat anti-mouse, Sample PMID: 31819006
Anti-rabbit IgG HRP-linked, Sample PMID: 36550132

Eukaryotic cell lines

Policy information about cell lines and Sex and Gender in Research

Cell line source(s)

HCT116 and HCT116 BAX-/- cells denoted as HCT116 BAX KO were originally from Bert Vogelstein's Lab (Zhang et al. 2000; DOI: 10.1126/science.290.5493.989). HCT116 BAX WT, HCT116 BAX P168G, HCT116 BAX E75K, MEF BAX WT, MEF BAX K21E, MEF BAX R134E and MEF BAX R145E stable cell lines were generated using retroviral transduction of HCT116 BAX KO and MEF BAX BAK DKO respectively with BAX-IRES-GFP. All other cell lines used in this study were purchased from ATCC and DSMZ. Leukemia cells: OCI-AML3 (DSMZ Cat. # ACC-582), U937 (ATCC Cat. # CRL-1593.2), NB4 (DSMZ Cat. # ACC 207), MOLM13 (DSMZ Cat. # ACC 554), HPB-ALL (DSMZ Cat. # ACC 483). Lymphoma cells: SUDHL-5 (ATCC Cat. # CRL-2958), Namalwa (ATCC Cat. # CRL-1432), SUDHL-16 (ATCC Cat. # CRL-2964). Non-small lung cancer cells: CALU-6 (ATCC Cat. # HTB-56). Colorectal cancer cells: COLO-320 (DSMZ Cat. # ACC 144), DLD1 (ATCC Cat. # CCL-221), RKO (ATCC Cat. # CRL-2577), HT29 (ATCC Cat. # HTB-38). Non-cancerous cells: BEAS-2B (ATCC Cat. # CRL-3588) and IMR90 (ATCC Cat. # CCL-186).

Authentication	Cell lines were authenticated by their vendor and the Albert Einstein College of Medicine Genomics Core Facility. Morphology, karyotyping, and STR profiling were performed to confirm the identity of human cell lines and to rule out both intra- and inter-species contamination.
Mycoplasma contamination	All cell lines tested negative for mycoplasma contamination.
Commonly misidentified lines (See ICLAC register)	No commonly misidentified lines were used in our study.

Plants

Seed stocks	N/A
Novel plant genotypes	N/A
Authentication	N/A
